# Enhancing physical activity in early childhood for 3–5-year-old children born very preterm: protocol for an Australian randomised controlled trial of Active-Prem

**DOI:** 10.1136/bmjopen-2026-124614

**Published:** 2026-11-02

**Authors:** Kate L I Cameron, Tara L FitzGerald, Sanne Peters, Free Coulston, Loni Binstock, Rheanna M Mainzer, Xinyang Hua, Ross A Clark, Jeanie L Y Cheong, Lex W Doyle, Peter J Anderson, Katherine J Lee, Kim Dalziel, Marguerite Lloyd, Jasmine Lloyd, Joy E Olsen, Gemma Duff, Prakriti Koirala, Gaela Kilgour, Stephanie Best, Alicia J Spittle

**Affiliations:** 1 Physiotherapy, The University of Melbourne, Melbourne, Victoria, Australia; 2 Clinical Sciences, Murdoch Children’s Research Institute, Melbourne, Victoria, Australia; 3 Melbourne School of Health Sciences, The University of Melbourne, Melbourne, Victoria, Australia; 4 Clinical Epidemiology and Biostatistics Unit, Murdoch Children's Research Institute, Melbourne, Victoria, Australia; 5 Paediatrics, The University of Melbourne, Melbourne, Victoria, Australia; 6 Melbourne School of Population and Global Health, The University of Melbourne, Melbourne, Victoria, Australia; 7 School of Health, University of the Sunshine Coast, Maroochydore, Queensland, Australia; 8 Newborn and Allied Health Research, The Royal Women’s Hospital, Melbourne, Victoria, Australia; 9 Obstetrics, Gynaecology, and Newborn Health, The University of Melbourne, Melbourne, Victoria, Australia; 10 School of Psychological Sciences, Monash University, Melbourne, Victoria, Australia; 11 Pediatrics, University of California, Irvine, California, USA; 12 Center for Newborn Research, Children’s Hospital of Orange County, Orange, California, USA; 13 Life’s Little Treasures Foundation, Melbourne, Victoria, Australia

**Keywords:** Exercise, Child, Community Participation, Randomized Controlled Trial, Implementation Science, HEALTH ECONOMICS

## Abstract

**Introduction:**

Children born very preterm (VPT: <32 weeks’ gestation) are less physically active than their term-born peers and face greater barriers to physical activity (PA). PA has many benefits and is important for children born VPT who are at increased risk for poor physical and mental health. This paper describes the protocol for a randomised controlled trial (RCT) evaluating the effectiveness of Active-Prem (a six-step intervention combining parent support, coach training and a term of community-based PA) compared with Goal Setting, at improving PA for children born VPT living in Australia.

**Methods and analysis:**

Type 1 hybrid effectiveness-implementation RCT involving 204 children born VPT (3–5 years’ corrected age) and their parent/caregiver. Active-Prem comprises: (1) setting PA participation goals; (2) online support for parents through the co-designed Active Start Active Prems programme; (3) matching children with an activity and a PA coach in their local community; (4) upskilling coaches through the co-designed Coaching Essentials: Premature Children in Sport programme; (5) an introduction session for children and families to their chosen activity and (6) one term (8–11 weeks) of the chosen activity, facilitated by a coach who has completed Coaching Essentials. Participants allocated to Goal Setting will complete step 1 only.

The primary outcome is PA measured using 7-day accelerometry at postintervention. Secondary effectiveness outcomes include parent-reported PA and goal attainment. Implementability outcomes include acceptability, feasibility, fidelity and cost-effectiveness. For effectiveness outcomes, mean differences or risk differences between groups will be estimated using linear regression for continuous outcomes or binomial regression for binary outcomes. Implementability outcomes will be summarised using descriptive statistics (quantitative measures) or thematic analysis (qualitative measures).

**Ethics and dissemination:**

This study has approval from the University of Melbourne Human Research Ethics Committee (2025-31093-64376-3). Findings will be disseminated through peer-reviewed publications, conference presentations, digital and print media and to participants.

**Trial registration number:**

ACTRN12625000183482.

**STRENGTHS AND LIMITATIONS OF THIS STUDY**

- This randomised controlled trial uses a hybrid design which allows a dual focus on effectiveness and implementation. Informed by the conceptual framework of implementability, assessment of feasibility, fidelity and acceptability increases the likelihood that the Active-Prem intervention is responsive to the preferences of the community it is targeting.
- All stages of this project included meaningful involvement from key knowledge holders, including individuals with lived experience of preterm birth, community providers of physical activity and healthcare professionals.
- Two components of the Active-Prem intervention were co-designed and the investigator team includes people with lived experience.
- Active-Prem is designed for potential larger scale implementation; it is delivered online and embedded within children’s local communities, enhancing the sustainability of both the intervention and the health behaviour (physical activity of children and their families/caregivers) by targeting families and community-based coaches.
- Although our primary outcome measure (accelerometry) is a valid tool for assessing children’s physical activity, collecting 7-day accelerometry data may be challenging for 3–5 years with sensory or behavioural difficulties.

## Introduction

 Preterm birth, or birth at <37 weeks’ gestation, accounts for approximately 10% of births worldwide. Of children born preterm, around 15% are born very preterm (VPT; <32 weeks’ gestation).^1^ At a group level, children born VPT in early childhood (aged 3–5 years) are less physically active than their term-born peers,^2^ with 50% not meeting Australian 24-hour physical activity (PA) guidelines.^2–4^ This is a major problem as poor PA during childhood has significant long-term health implications including increased chronic disease burden and healthcare costs.^5 6^ For children born VPT, this is particularly concerning as they are at greater risk of poorer physical health outcomes, including worse cardiovascular^7^ and metabolic health,^7^ lower bone density^8^ and decreased lung function^9^ than children born at term, with disparities continuing into adulthood.^7^ Furthermore, children born VPT are at greater risk of adverse developmental outcomes compared with children born at term, including motor impairment (cerebral palsy or developmental coordination disorder),^10 11^ cognitive impairment, behavioural/social challenges^12^ and poorer mental health.^13^

PA is associated with a range of health and developmental benefits for all children, including better motor skills, greater physical fitness, improved cognitive outcomes, lower rates of chronic disease and better academic outcomes.^5 14 15^ Furthermore, PA may improve confidence and promote enjoyment and quality of life.^16^ Thus, drawing from the broader literature on the benefits of PA for children with and without disabilities, PA offers a multifaceted intervention option for children born VPT given its positive association with outcomes across multiple developmental and health domains that are known to be vulnerable following VPT birth.^7 9–12^ PA in early childhood is also associated with higher PA in later childhood and adulthood,^17^ making early childhood an opportune time to intervene. Despite this, there are currently few interventions aimed at improving PA for children born VPT during this pivotal developmental period.^18^

The family of participation-related constructs (fPRC) is a framework that provides a clear definition of participation as both attendance (being physically present) and involvement (the experience of participating).^19^ The fPRC describes a bidirectional relationship between participation and both the participating child and the context and environment in which the activity takes place.^19^ Participation in PA as an intervention mechanism has potential to improve PA while taking context into account, such as through targeting environmental and social factors that influence PA outcomes in children, such as family involvement.^20 21^ Randomised controlled trials (RCTs) that explore PA or participation for 3–5-year-old children, however, are scarce.^19^

Active-Prem is a novel intervention built on substantial preliminary research into understanding and improving PA participation for 3–5-year-old children born VPT, and addresses an expressed need from parents of children born VPT for support with PA participation for this age group.^2–4 22–25^ It uses participation as a means; working with children and families to support attendance and involvement, taking into account preferences and family contexts, and directly targeting the environment through parent support and coach training. Active-Prem aims to address the greater barriers to participation experienced by children born VPT compared with their term-born peers, including difficulty accessing physical activities and lack of knowledge and awareness of preterm birth in the community.^3 4 26^

Active-Prem addresses the urgent need for trials to address the PA gap by embedding interventions into families’ existing local community offerings (such as children’s dance, gymnastics, swimming, soccer and multisport classes) and scaffolding children’s meaningful participation via family support and upskilling of activity providers (coaches). Further, to enhance implementability, and the likelihood that an intervention will be scalable and sustainable,^27^ it is important to leverage existing resources and provide real world PA participation opportunities in families’ local environments.

This paper describes the protocol for an RCT that aims to evaluate the effectiveness and implementability of Active-Prem to improve PA for 3–5-year-old children born VPT. Active-Prem comprises six key steps: (1) setting PA participation goals; (2) providing online support for parents through the co-designed Active Start Active Prems programme; (3) matching children with an activity (based on their goals) and a community PA provider (coach) in their local community; (4) upskilling coaches through the co-designed Coaching Essentials: Premature Children in Sport programme; (5) an introduction session for children and families to their chosen activity and (6) one term (8–11 weeks) of PA facilitated by a coach who has completed Coaching Essentials.

### Objectives

The primary objective is to determine the effectiveness of Active-Prem compared with Goal Setting at improving objectively measured PA for children aged 3–5 years’ corrected age (CA) born VPT postintervention. Secondary objectives are described in table 1.

**Table 1:** Secondary objectives

Objective type	Objective
Effectiveness	a. To determine the effectiveness of Active-Prem compared with Goal Setting at improving objectively measured PA at 12 months post-randomisation parent-reported PA at postintervention and 12 months post-randomisation for children aged 3–5 years’ CA born VPT.
Effectiveness	b. To determine the effectiveness of Active-Prem compared with Goal Setting at improving PA participation goal attainment postintervention for children aged 3–5 years’ CA born VPT. Each participant will set 3 goals. Goals need to include 1× attendance, 1× involvement and 1× attendance or involvement or sense of self.
Effectiveness	c. To determine the effectiveness of Active-Prem compared with Goal Setting at improving parent-reported child behaviour for children aged 3–5 years’ CA born VPT post-intervention and 12 months post-randomisation.
Implementability	d. To determine the implementability of Active-Prem including measures of acceptability, feasibility and fidelity from the perspectives of participating children, their parents and coaches (intervention group only).
Effectiveness	e. To determine the effectiveness of the Active Start Active Prems parent programme compared with Goal Setting at improving parent perceived barriers and facilitators to supporting their child’s community-based PA participation at postintervention.
Effectiveness	f. To determine the effectiveness of the Coaching Essentials: Premature Children in Sport programme at improving coaches’ knowledge, skills and confidence to successfully and meaningfully include children born preterm into their preschool-aged classes (from baseline to postintervention). Note: all coaches are in the intervention group.
Cost-effectiveness	g. To determine the cost-effectiveness of Active-Prem compared with Goal Setting measured using an incremental cost-effectiveness ratio postintervention and at 12 months post-randomisation.
Definitions of measurement time points:	
The intervention period occurs across two preschool/school terms. Baseline: A ‘typical week’ prior to randomisation. Postintervention: A ‘typical week’ after the intervention period has concluded (eg, at the end of the preschool/school term). 12 months post-randomisation: A ‘typical week’ that occurs 12 months post-randomisation. A typical week is defined as a week during which a parent reports that the child is participating in their usual activities and usual routines (eg, not a week when the family are on holiday or when routines are altered for other reasons).	

PA, physical activity; CA, corrected age; VPT, very preterm.

## Methods and analysis

### Trial design

This study is an RCT with a type 1 effectiveness-implementation hybrid design.^28^ The Active-Prem intervention will be compared with a Goal Setting intervention. We have chosen to have a comparison intervention rather than wait list due to the potential bias towards the tested intervention in exercise interventions with wait-list control design.^29^ Effectiveness-implementation designs aim to reduce the time lag between research and practice to enable more rapid translation of evidence, by considering both effectiveness and the likelihood of future implementation success within the same trial design.^28^ We have chosen a type 1 design, in which the effectiveness of the intervention is the primary aim, while secondary aims gather information on intervention delivery and its potential for implementation in a real-world situation. This protocol is reported according to the Standard Protocol Items: Recommendations for Interventional Trials- Children and Adolescents (SPIRIT-C) guidelines^30^ and the Template for Intervention Description and Replication (TIDiER) checklist,^31^ and in adherence to the National Statement and Australian Code for Responsible Research Conduct. This trial is registered with the Australian New Zealand Clinical Trial Registry (ACTRN12625000183482).

### Participants

There are two participant groups in this trial:

1. Children and a parent/caregiver of each child.
2. Coaches of community-based physical activities.

### Children born VPT and their parent/caregiver

Children and a parent/caregiver of each child will be enrolled in the study as a parent–child dyad.

#### Inclusion criteria

1. Children born <32 weeks’ gestation.
2. Children aged 3–5 years (CA) at the time of enrolment in the study.
3. Children able to independently ambulate, with/without aids or orthoses.
4. Child and their parent/caregiver are planning to reside in Australia for the intervention duration.
5. Parent/caregiver age ≥18 years at the time of enrolment.
6. Parent/caregiver able and willing to complete the online Active Start Active Prems parent programme delivered in English.
7. Child and their parent/caregiver are available to participate in the intervention programme for a 6-month period.

#### Exclusion criteria

Children who have any medical condition, or other health-related reason, that precludes safe participation in PA.

Children with planned medical procedures during the intervention period that precludes safe participation in PA.

This study aims to recruit as inclusively as practicable. The focus is on safety rather than excluding children with medical conditions who may require guidance from a healthcare professional to participate. For children whose parent/carer indicate that their child may not be safe to participate, a member of the study team will (with the permission of the parent/carer) seek the opinion of the child’s treating medical professional as to the safety of the child’s participation. Written permission will be sought from treating healthcare professionals in each of these cases. For children who have a medical procedure planned, the study team will seek permission to re-contact the family at a later date (if the study timeline allows) to re-evaluate eligibility.

### Coaches

Coaches will be recruited on a case-by-case basis for each parent-child dyad allocated to the Active-Prem arm of the RCT. Therefore, all coaches will participate in the Active-Prem intervention.

#### Inclusion criteria

1. Coaching a regularly scheduled (at least 1/week) PA class for 3–5-year-old children that can be reasonably expected to run for the duration of the intervention period.
2. Coaching in Australia.
3. Able and willing to complete the online Coaching Essentials: Premature Children in Sport programme delivered in English.
4. Have at least 1 year of experience coaching PA, or equivalent (eg, assistant coaching, disability/teaching role) to preschool-aged children.
5. Have a valid Working with Children Check.
6. Have a valid First Aid Certificate (or a staff member will be present during the activity who is the designated first aider).

#### Exclusion criteria

1. Coach age <18 years at the time of recruitment.

### Recruitment and informed consent

Recruitment for Active-Prem commenced in April 2025 and is planned to be completed by September 2027. Recruitment was initially restricted to the state of Victoria, Australia. However, in November 2025, recruitment was expanded to Australia-wide.

#### Parent–child dyads

Families will be recruited from across Australia via advertising through our partner organisations (eg, Life’s Little Treasures Foundation and Miracle Babies) who support families of children born preterm, through organisations affiliated with our research team (The University of Melbourne, The Royal Children’s Hospital, Royal Women’s Hospital and Murdoch Children’s Research Institute), sharing advertising materials among clinical Australian paediatric physiotherapists and social media platforms that target parents of children in early childhood. Advertising will include printed flyers and bookmarks, social media posts and presence at partner organisation events.

Potential participants will be asked to complete an online expression of interest form to screen for eligibility. Eligible families will be contacted by a member of the research team, provided with written information about the study and offered an opportunity to discuss the project and ask questions. All members of the research team are trained in cultural safety and awareness to facilitate the inclusion of First Nations families. If families agree to participate, parents will be asked to sign a consent form on behalf of themselves and their child/children (online supplemental file A).

#### Coaches

Coaches will be recruited via the same advertising strategies listed above. In addition, coaches will be approached via email or phone call (using publicly available contact information) to match to a participating child. Recruiting community coaches on a per-child participant basis has been successfully achieved for preschool-aged children born VPT in our pilot project.^22^ Coaches will also be asked to sign a consent form (online supplemental file A).

### Description of interventions

#### Intervention: Active-Prem

The Active-Prem intervention comprises six key steps (figure 1), each described using the TIDiER format: items 1–3 (name, rationale and ‘what’) are described below, while the remaining items (‘who’, ‘how’, ‘where’, ‘when and how much’ and ‘tailoring’) are detailed in table 2.^31^

**Figure 1:**
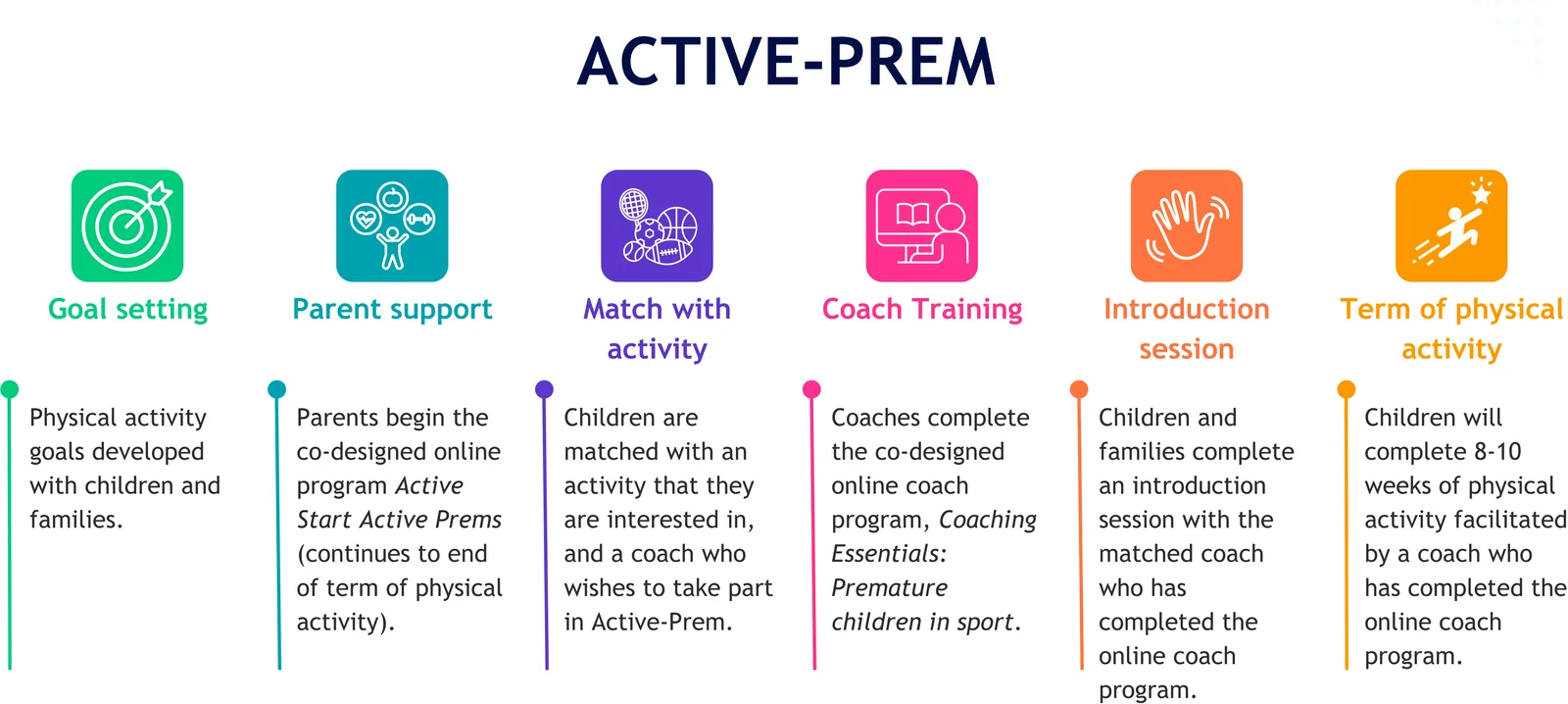
Six steps of Active-Prem intervention.

**Table 2:** Description of Active-Prem using the Template for Intervention Description and Replication (TIDieR) checklist

TIDieR items					
Active-Prem step	Who	How	Where	When and how much	Tailoring
Step 1: Goal setting	A physiotherapist or occupational therapist trained in the COPM and blinded to group allocation.	A motivational interview conversation format.	Video conference or phone call.	During the baseline assessment period.	Goal setting will be individualised. Children have the option to be included in goal setting.
Step 2: Parent support: Active Start Active Prems parent programme	Written and presented by experts in each content area: parents of children born VPT, coaches, physiotherapists, occupational therapists and psychologists.	Website using a range of mediums; written and visual content, short videos and mini podcasts. Peer support sessions will be delivered via online teleconferencing.	Delivered online to promote accessibility and scalability.	Starts after randomisation and continues until the completion of step 6. Self-directed learning=5 hours. Peer support=one ‘Welcome to Active-Prem’ online session (1 hour; compulsory) and two subsequent check-in sessions during step 6 (1 hour; optional).	Optional aspects include module addressing common concerns and worries, participation in discussion board and 2× check-in sessions.
Step 3: Matching with an activity	Matching will be facilitated by an unblinded researcher.	Our research team will recruit coaches on a 1:1 basis.	Not applicable.	Matching will occur prior to steps 4–6.	Matching will be based on the children’s and family’s PA preference.
Step 4: Coach training: Coaching Essentials: Premature Children in Sport programme	Written and presented by experts in each content area: parents of children born VPT, coaches, physiotherapists, occupational therapists, speech pathologists and psychologists.	Delivered online; self-directed learning will be delivered via an online educational platform and optional coach support workshops will be delivered via teleconferencing.	Delivered online to promote accessibility and scalability.	Open to coaches from consent until completion of step 6. Coaches will need to complete the self-directed components and associated assessments of Coaching Essentials prior to step 5. The self-directed learning contains 8 hours of content (4 modules). The optional coach support workshops will be 1 hour long and are offered 6–8 times across the year.	Self-directed learning is designed to be completed within approximately 4 weeks. However, this duration can be flexible based on individual preferences and circumstance. Coach support workshops will be optional, and coaches can choose to attend as many of them as they feel they need (titrating their own support).
Step 5: Introduction session	Delivered by the participating matched coach.	Face-to-face. Children and parent/caregiver with their matched coach.	At the location of their chosen PA (eg, at the sports club).	Prior to step 6. Approximately 60 min in duration; includes ‘getting to know you’ time and a tailored introduction to the activity.	Individualised depending on the needs and preferences of families/coaches
Step 6: Term of physical activity (subsidised by the study to minimise financial burden)	Classes will be delivered by a coach who has completed Coaching Essentials: Premature Children in Sport.	Face-to-face group PA class.	At the location of their chosen activity (eg, at the sports club).	Once per week for the duration of a preschool term (typically 8–11 weeks).	Activity selected based on child and family preference. Families may choose to continue after the study has concluded

COPM, Canadian Occupational Performance Measure; PA, physical activity; VPT, very preterm.

#### Step 1) Goal setting (note: this step is completed by both Active-Prem and Goal Setting groups)

Why? (rationale): goal setting is recognised as an important and effective strategy for behaviour change including interventions promoting participation in PA for children.^32^

What: goals will be set using the Canadian Occupational Performance Measure (COPM).^33^ Goals will aim to improve PA and will align with the fPRC key concepts; one ‘attendance’ goal, one ‘involvement’ goal and one ‘attendance’ or ‘involvement’ or ‘sense of self’ goal.^19^

#### Step 2) Parent support: Active Start Active Prems parent programme

Why? (rationale): the Active Start Active Prems programme has been co-designed and is intended to address key barriers to and facilitators of PA participation for children born VPT, identified from previous research and prioritised through the co-design process.^3 4 23 34 35^

What: this co-designed programme aims to support parents to better support their child’s participation in community-based PA. The programme consists of five self-directed learning modules (one of which is optional) covering topics such as ‘The benefits of PA for children born VPT’, ‘Choosing activities to suit the child’, ‘Preparing for the introduction session’, ‘Modelling and goal setting’ and ‘Communicating about your child in a recreational PA setting’, along with tools and strategies for supporting the child’s PA journey. The programme also includes a PA ideas library, peer support discussion board and group ‘check-in’ sessions.

#### Step 3) Matching with an activity

Why? (rationale): children are more likely to engage and participate in physical activities that match their preferences and are fun.^23 36 37^ The Active-Prem intervention will use existing local community PA offerings to enhance social connections within the family’s local community, thus is designed to facilitate real-world implementation and may increase the sustainability of the PA participation opportunities within local environments.

What: children will be individually matched with a coach in their local community. Matched coaches will need to be eligible and willing to participate in Active-Prem as a coach.

#### Step 4) Coach training: Coaching Essentials: Premature Children in Sport programme

Why? (rationale): lack of knowledge of preterm birth and skills for including children with disabilities have been identified as significant barriers to PA participation for children born VPT.^4^ The Coaching Essentials: Premature Children in Sport programme has been co-designed and builds on two previously tested programmes (both developed by our research team) aiming to upskill community providers of PA; Dance PREEMIE (dance teachers)^22 23 38^ and CirqAll (circus coaches).^39^

What: the overall goal of this programme is to improve coaches’ knowledge, skills and confidence to better include children born VPT in their classes. The programme includes self-directed learning and optional Coach Support Workshops. Self-directed learning includes knowledge content, tasks and assessments. Topics covered in this content include: ‘understanding prematurity’, ‘understanding key outcomes for children born prematurely’, ‘supporting physical skill development’, ‘supporting social, emotional and behavioural skill development’, ‘developing goals and lesson objectives’ and ‘communication with the team around the child’. Coach Support Workshops are designed to provide a supportive space to problem-solve challenges across the Coaching Essentials course and/or Active-Prem project, and to facilitate a community of practice. Coaches are provided with an $A200 gift-voucher to acknowledge the time taken to complete the training.

#### Step 5) Introduction session

Why? (rationale): this session is designed to address barriers including coach knowledge of individual children’s support needs and strengths, and parent knowledge of expectations of the activity and how they can best prepare their child.^39^

What: children and parents will attend an introduction session with their matched coach, allowing parents and children to meet the coach, discuss the child’s individual support needs and become familiar with the environment and activity. Coaches and families will be supported to prepare for this session as part of steps 2 and 4 respectively. The cost of the 1:1 introduction session will be subsidised by the study to minimise financial burden for families.

#### Step 6) Term of PA

Why? (rationale): integrating the Active-Prem intervention into already existing PA classes in the local community (ie, ‘mainstream’ or ‘open to the public’ classes) enhances the likelihood that improved PA participation will be sustainable for children and families. Furthermore, this approach is more likely to be implementable and scalable (if the intervention is effective) as it can be embedded into local communities. This approach has been successfully trialled in a feasibility study using dance classes.^22^

What: children will attend one school term of their chosen activity in a publicly available PA class, facilitated by their matched coach. One term of the activity will be subsidised by the study to minimise financial burden for families.

### Control: Goal Setting

The Goal Setting group will complete step 1 of the Active-Prem intervention with a physiotherapist or occupational therapist. There is no ‘standard care’ for improving PA for 3–5 years’ CA children born VPT, however goal setting is a commonly used and evidenced-based approach.^32^

### Both groups

The following are behaviour change strategies which will be provided to families in both the Goal Setting and Active-Prem groups as part of the study.

- Families will be given a copy of the Australian 24-hour Movement Guidelines: birth to 5 years brochure, a publicly available resource outlining PA recommendations for this age group.^40^
- All participants will be given two reports: one after the postintervention assessment period and one at the conclusion of the study (after the 12 months post-randomisation assessment). The reports will include information on whether each child is meeting the 24-hour Movement Guidelines (based on PA outcome measures), as well as changes to the child’s PA over time.
- Goal setting is a recognised behaviour change strategy for improving PA, and is completed at baseline by both groups.

### Enrolment

The intervention period takes place over two preschool/school terms (Active-Prem steps 1–5 in the first term, and step 6 in the subsequent term). Children will therefore be enrolled to time with school terms.

### Data collection, management and access

Data for this trial will be collected using electronic data collection forms using The University of Melbourne’s Research Electronic Data Capture (REDCap) database system, which will be completed either directly by the participant (parent or coach) or by a member of the research team. Objective PA data will be collected using triaxial accelerometry.

### Baseline data collection

#### Accelerometry

PA will be assessed with a high-resolution (13-bit), water resistant to 1.5 m, ankle mounted triaxial accelerometer (Axivity AX3, Newcastle on Tyne, UK) worn for seven consecutive days (including a full weekend) for 24 hours/day. Ankle-mounted accelerometers have been used previously to assess PA for preschool-age children born VPT^2^ and the ankle is well established in research as the optimal position for stepping movements across a range of devices.^41 42^ Axivity AX3 accelerometers have been found to be a valid tool for assessing walking and running in preschool-aged children.^43^ The Axivity AX3 accelerometer has been chosen as it allows for collection of raw acceleration data, which facilitates the transparent reporting of accelerometer data management protocols by avoiding the use of proprietary algorithms. Our team has prior experience using custom-built software for accelerometer processing. 7-day accelerometry will also be collected postintervention and 12 months post-randomisation. Accelerometer-measured outcomes account for the spectrum of movement behaviours over 24-hours and include: (1) total PA (minutes/day) determined by summing each non-stationary minute during waking hours; (2) time (minutes) spent stepping per day (stepping PA, primary outcome); (3) total number of steps per day; (4) moderate-vigorous PA, determined by summing the time spent in fast steps per day (minutes) and (5) stationary time (minutes/day) obtained by summing each minute with no recorded steps during waking hours.^44^ Further details on accelerometer data management are included in online supplemental file B.

The COPM^33^ is a valid and reliable goal setting tool that uses a semistructured interview format to identify and prioritise child-centred and meaningful goals.^45^ A goal setting conversation will be completed with parents/caregivers and a physiotherapist or occupational therapist familiar with using the fPRC framework and trained in administering the COPM. Children will be encouraged to join the goal setting conversation if they wish. Families will be supported to set three PA goals related to participation constructs as defined by the fPRC;^19^ one ‘attendance’ goal, one ‘involvement’ goal and one ‘attendance’ or ‘involvement’ or ‘sense of self’ goal. Goals are scored on two scales: performance and satisfaction—each scored from 1 (lowest score) to 10 points (highest score), with a change of 2 or more points considered clinically significant.^45^ Goals are rescored postintervention.

Parent-report questionnaire outcome measures collected at baseline are presented in table 3.

**Table 3:** Baseline parent-report questionnaires

Demographic questionnaire	A brief demographic questionnaire (online supplemental file D) will ask about the participating child’s birth history (including gestational age), diagnoses and current access to allied health interventions (eg, physical therapy). The questionnaire will also ask about aspects of socioeconomic status, including postcode. This questionnaire will take approximately 5 min to complete.
Little Developmental Coordination Disorder Questionnaire (Little DCDQ) or the Developmental Coordination Disorder Questionnaire (DCDQ-’07)	The Little DCDQ is a parent-report questionnaire designed as a screening tool for motor coordination difficulties in 3–4-year-old children,^54^ while the DCDQ-’07 is a parent-report questionnaire screening tool for motor coordination difficulties for 5–15-year-old children.^55^ Families will be provided with the questionnaire appropriate for the age of the participating child.
Strengths and Difficulties Questionnaire (SDQ)	The SDQ is a valid and reliable parent-report questionnaire that assesses children’s overall behaviour including emotional symptoms, conduct problems, hyperactivity/inattention, peer relationships and prosocial behaviours (25 items in total).^56^ Also collected post-intervention and 12 months post-randomisation.
Community section of Young Children’s Participation and Environment Measure (YC-PEM)	The YC-PEM is a valid parent-report questionnaire exploring children’s participation in community activities, including frequency of participation, involvement in the activity and parent satisfaction with their child’s participation. The YC-PEM also explores the impact of the environment on participation in community activities.^57^
Child Health Utility 9D (CHU9D)	The CHU9D is a valid health-related quality of life measure for children, with parent-report versions now available for children under 7 years. Also collected post-intervention and 12 months post-randomisation.
Movement Behaviour Questionnaire Child Open Version (MBQ-C)	The MBQ-C is a valid questionnaire for measuring active play, screen time and sleep (all components of the 24-hour movement guidelines) in children aged 0–5 years.^58^ Some additional questions have been added to the MBQ-C for the purposes of our study to understand children’s current community-based PA. (online supplemental file D) Also collected post-intervention and 12 months post-randomisation.
Barriers and facilitators for supporting children’s community-based physical activity participation	A series of open-ended questions developed for this study will explore parent-perceived barriers and facilitators to supporting their child’s PA participation (online supplemental file D). These questions have been developed with input from the implementation scientists in our team and further refined through a ‘think aloud’ interview process with parents involved in our study.^59^ Also collected postintervention.
International Physical Activity Questionnaire (IPAQ)	The IPAQ is a standardised self-reported measure of PA for adults.^60 61^ The IPAQ will be used to measure parental/caregiver PA at baseline, postintervention and 12 months post-randomisation.

PA, physical activity.

### Randomisation

Stratified block randomisation (with variable block sizes) will be used to allocate parent-child dyads 1:1 to the Active-Prem or Goal Setting groups using a web-based randomisation system. Randomisation will take place following informed consent and after baseline measures have been completed. Randomisation will be stratified by whether the family has one child versus more than one child enrolling in the study, with children in the same family randomised to the same group. This approach was chosen so that all children in the same family receive the same intervention and because parents of children randomised to Active-Prem complete the parent programme as part of the intervention. Randomisation lists will be generated in Stata by an independent statistician. Preintervention measures after group allocation are outlined in table 4.

**Table 4:** Preintervention measures after group allocation

Participant group	Outcome measure
Families (Active-Prem group only)	Theoretical Framework of Acceptability (TFA) Survey: Parent-perceived acceptability of Active-Prem, measured using the TFA Survey.^62^
Coaches	TFA survey: Coaches-perceived acceptability of Active-Prem will be measured using the TFA Survey.^62^
	Demographics questionnaire: A brief demographic questionnaire will ask about which activity the coach teaches, their relevant qualifications and experience (online supplemental file E).
	Determinants of Implementation Behaviour Questionnaire (DIBQ): Implementation behaviours of coaches will be measured using the DIBQ, a valid questionnaire based on the Theoretical Domains Framework.^63^ This questionnaire has been adapted (as it is designed to be) to focus on the target behaviours of coaches.

### Outcome measures

The primary outcome is minutes spent stepping per day (averaged over 7 days) measured by accelerometry at postintervention. Secondary outcome measures are outlined in table 5, while a schedule of assessments and events is provided as online supplemental file C. Outcome measures developed specifically for the study are included as online supplemental file D (parent–child outcomes) and online supplemental file E (coach outcomes).

**Table 5:** Secondary outcome measures

Secondary outcomes	Outcome measures and timepoints
Effectiveness of Active-Prem compared with Goal Setting	Objectively measured PA using 7-day accelerometry (average minutes per day spent stepping) at 12 months post-randomisation (secondary objective a).
	Parent-report PA measured using the MBQ-C and additional open-ended questions (online supplemental file D) at postintervention and 12 months post-randomisation (secondary objective a).
	Change in participation in PA goal satisfaction and performance measured using the COPM at post-intervention compared with baseline (secondary objective b).
	Parent-reported child behaviour measured using the SDQ at post-intervention and 12 months post-randomisation (secondary objective c).
Acceptability of the Active-Prem intervention (Active-Prem group only, secondary objective d)	Parent-perceived acceptability of the Active-Prem intervention measured using a survey based on the TFA^62^+additional study-specific open-ended questions at postintervention.
	Child-perceived acceptability of the Active-Prem intervention measured using a smiley face scale at the conclusion of each class.
	Coach perceived acceptability of the Active-Prem intervention measured using a survey based on the TFA^62^+additional open-ended questions at post-intervention.
Fidelity of Active-Prem (Active-Prem group only, secondary objective d)	Fidelity of the classes will be assessed using the Fidelity and Adaptability survey (online supplemental file E) completed by coaches after class #4 and class #8.
	Fidelity of the Active Start Active Prems parent programme will be assessed using engagement rates from website platform.
	Fidelity of Coaching Essentials: Premature Children in Sport will be assessed using engagement rates from the online educational platform as well as completion of compulsory learning tasks.
Feasibility of Active-Prem (secondary objective d)	Feasibility measured through a file audit (# of children who participated/ # eligible, attendance rate of children, # of coaches who participated; # eligible, number of drop-outs) throughout trial.
	Implementation barriers and enablers from the perspectives of parents and coaches will be evaluated using semistructured interviews (based on the Theoretical Domains Framework)^64^ with a subset of purposively sampled participants post-intervention.
Effectiveness of Active Start Active Prems (Active-Prem group only)	Parent-perceived barriers and facilitators to supporting their child’s participation in community-based PA measured using study specific open-ended questions (online supplemental file D) at postintervention (secondary objective e).
Effectiveness of Coaching Essentials: Premature Children in Sport at improving coaches’ knowledge, skills and confidence to include children born VPT in their classes	Coach-perceived behaviour measured using the DIBQ at postintervention compared with baseline (secondary objective f).
Cost-effectiveness of Active-Prem compared with Goal Setting (secondary objective g).	Child health-related quality of life measured using the CHU9D^65^ at baseline, postintervention and 12 months post-randomisation.
	Costs of the intervention estimated through study protocols and interviews with personnel delivering aspects of the interventions.
	Downstream costs estimated through family report of healthcare utilisation 12 months post-randomisation.
	Cost-effectiveness calculated as the incremental cost-effectiveness ratio comparing Active-Prem to Goal Setting with a 1-year horizon.

CHU9D, Child Health Utility 9D; MBQ-C, Movement Behaviour Questionnaire Child; PA, physical activity; TFA, Theoretical Framework of Acceptability; VPT, very preterm.

### Blinding

Participants, including coaches, children and their families cannot be blinded due to the nature of the intervention. Outcome measures (effectiveness outcomes) at postintervention and 12 months post-randomisation will be collected by assessors blinded to intervention allocation. All assessors will be trained in the protocol to ensure consistency between assessors. Processing of accelerometry data (primary outcome) and data management for all effectiveness outcome measures, will be completed by a research team member blinded to intervention allocation. Acceptability outcome measures will only be completed by participants allocated to the Active-Prem group, so blinding will not be possible.

### Sample size calculation

As we are conducting a type 1 hybrid design, the study will be powered based on the intervention effectiveness primary outcome: minutes spent stepping per day (averaged over 7 days) measured by accelerometry postintervention. This study will require 204 children in total (approx. 102 per arm) to have 80% power to detect a mean difference of 30 min of stepping per day between the Active-Prem and Goal Setting groups using a two-sided test with alpha=5%, a difference considered to be clinically meaningful. This calculation assumes the SD of time spent stepping per day is 64 min in each arm based on our previous research,^1^ allows for 20% drop out and accounts for an expected 25% of children being multiples (twins/triplets) with an intracluster correlation of 0.80.^46^

### Data analysis

A detailed statistical analysis plan (SAP) will be finalised and made publicly available prior to initiating the analysis at the conclusion of the trial. Further details on data management are included in online supplemental file B.

#### Baseline characteristics

Baseline characteristics for children and families, including data collected in the demographic questionnaire and from outcome measures only collected at baseline, will be presented using descriptive statistics by intervention group. Baseline characteristics for coaches, including data from the demographic questionnaire, will be presented using descriptive statistics.

#### Analysis of primary objective

The primary estimand of interest is the mean difference in average minutes spent stepping per day measured by accelerometry during the postintervention period, between the Active-Prem and Goal Setting groups, using a treatment policy approach (all participants will be analysed according to their treatment allocation irrespective of whether or not they received any of the intervention) to handle the intercurrent event of non-compliance to the intervention.^46^ This will be estimated using linear regression, adjusted for multiple birth. The model will be fitted using generalised estimating equations to account for the correlation between siblings (eg, twins) within families and robust (sandwich) SEs will be used to estimate the 95% CI.

Supplementary analyses will explore the efficacy of the intervention using a hypothetical strategy to handle non-compliance in the Active-Prem group. To achieve this, participants not complying with the intervention will have their outcome set to missing, and multiple imputation will be used to handle the missing outcomes.

For both analyses we will explore whether the effect of the intervention varied between children attending school versus preschool by including an interaction term in the analysis model, although we acknowledge that this analysis will be underpowered, and the results will be interpreted with caution.

#### Analysis of secondary objectives

##### Effectiveness

The same approach described for the primary analysis of the primary outcome (using a treatment policy approach to handle intercurrent events) will be used to analyse minutes spent stepping per day at 12 months post-randomisation to assess whether the effects of the intervention are sustained. Similar analyses will be carried out to estimate the effect of the intervention on parent-reported PA measured using the Movement Behaviour Questionnaire Child Open Version (MBQ-C) and PA participation goal attainment measured using the COPM, both post-intervention and at 12 months post-randomisation (MBQ-C only).

Effectiveness of the Active Prem Coaching Essentials: Premature Children in Sport programme at improving coach behaviour to successfully and meaningfully include children born VPT in their classes, measured using the Determinants of Implementation Behaviour Questionnaire (DIBQ), will be summarised using descriptive statistics and visual representations in the intervention group. Likewise, parent perceptions of barriers and facilitators to supporting their child’s PA will be analysed descriptively and compared between the Active-Prem and the Goal Setting groups. Estimated intervention effects for secondary outcomes will be interpreted with caution as the study is powered for the primary outcome.

##### Implementability

Quantitative measures of acceptability, feasibility and fidelity will be summarised using descriptive statistics (data from intervention group and coaches only). Frequencies and percentages will be presented for categorical data, for example, the acceptability of Active-Prem.

The intervention will be considered to have high acceptability if ≥80% of respondents rate the intervention ‘acceptable’ or ‘very acceptable’ on the Theoretical Framework of Acceptability (TFA) survey, moderate acceptability if 50% to 79% of respondents rate the intervention ‘acceptable’ or ‘very acceptable’ on the TFA, or low acceptability if <50% of respondents rate the intervention ‘acceptable’ or ‘very acceptable’ on the TFA, based on overall TFA scores.

Acceptability from the perspectives of participating children will be assessed using a Smiley Face Scale (5-point Likert scale). The intervention will be considered to have high acceptability if ≥80% of respondents rate their activity classes ‘really good’ or ‘fantastic’ (score of 4 or 5) on the Smiley Face Scale, moderate acceptability if 50% to 79% of respondents rate their activity classes ‘really good’ or ‘fantastic’ on the Smiley Face Scale, or low acceptability if <50% of respondents rate their activity classes ‘really good’ or ‘fantastic’ on the Smiley Face Scale, based on overall scores.

Fidelity of the intervention will be defined as high (adherence of 80%–100%), moderate (50%–79%) or low (<50%).^47^

Feasibility will be measured via a file audit of recruitment, attendance rates and dropout rates (throughout the study). Feasibility will be considered in regard to attendance rates and retention in the study as: highly feasible (80%–100%), moderately feasible (50%–79%) and low feasibility (<50%).

Qualitative data (eg, open-ended survey questions) will be analysed using reflexive thematic analysis,^48^ with results presented as themes.

##### Cost-effectiveness

The economic evaluation will be conducted from the healthcare sector perspective with a 1-year time horizon and reported following the Consolidated Health Economic Evaluation Reporting Standards 2022.^49^ The incremental cost-effectiveness ratio (ICER) will be calculated as the cost per quality-adjusted life-year gained for the Active-Prem compared with the Goal Setting group and will be compared with a willingness-to-pay threshold of A$50 000 to determine the cost-effectiveness of the intervention. ICERs for some other secondary outcomes will also be explored, for example, cost per additional child meeting PA guidelines. A bootstrap method with 1000 replications will be used to generate the 95% CI of the ICER and a cost-effectiveness plane.

##### Missing data

Causal diagrams will be used to depict assumptions regarding the causes of any missing data and guide the analytic approach to handle the missing data,^50^ for example, using multiple imputation to handle missingness if deemed appropriate. Further details of this process will be outlined in a SAP which will be finalised and made publicly available prior to initiating the analysis.

### Patient and public involvement

To develop the study question, funding application and protocol for this study, we have engaged and partnered with three groups of key knowledge holders: (1) individuals with lived experience of preterm birth, (2) community providers of PA and (3) healthcare professionals working with children born VPT. Specifically, we have engaged and consulted with leading consumer groups (Life’s Little Treasures Foundation, Miracle Babies Foundation) as well as community PA providers (AllSportz, Gymnastics Victoria, Kinderballet, Kelly Mini Sports and The Circus Spot). We also consulted with the Consumer Advisory Group of the Centre of Research Excellence (CRE) in Newborn Medicine. Three people with lived experience of preterm birth were listed as investigators on our funding application and are authors on this protocol (LB, ML and JL).

The Active-Prem intervention includes two co-designed components: the Active Start Active Prems (step 2) and the Coaching Essentials: Premature Children in Sport (step 4). Each co-design group (Active Start Active Prems n=9; Coaching Essentials n=10) comprised parents of children born preterm, healthcare professionals and coaches, and was facilitated by a researcher in the field of preterm birth and a parent with lived experience. Each programme was tested for acceptability through focus groups involving additional knowledge holders (Active Start Active Prems n=31; Coaching Essentials n=26).

In addition, this project has an Advisory Committee which provides independent oversight and ensures that the Active-Prem study is conducted in line with co-constructed values. The Advisory Committee is made up of knowledge holders who are not directly involved in other aspects of the study, including parents of children born VPT or representatives of preterm support groups (n=3), healthcare professionals (n=2) and community PA providers (n=2).

## Ethics and dissemination

### Ethics

This study has approval from the University of Melbourne Human Research Ethics Committee (2025-31093-64376-3). Safety oversight will be under the direction of an independent Data and Safety Monitoring Board (DSMB). Further details on the DSMB, trial management, risk and safety are provided in online supplemental file B.

### Dissemination

Trial findings will be disseminated through publication in peer-reviewed journals, presentations at scientific conferences, as well as digital and print media. In addition, research results will be shared with study participants, individuals with lived experience of preterm birth, health professionals and PA providers. This will be facilitated through direct communication with participants, collaboration with parent support groups and PA provider organisations using social media and web platforms, as well as through the Extremely Preterm Infant CRE. If found to be effective, Active-Prem will be implemented more broadly using this protocol, or adapted from the protocol based on the acceptability, feasibility and fidelity findings of this study.

## Discussion

This paper describes the protocol for hybrid effectiveness-implementation trial of a PA participation intervention (Active-Prem) compared with Goal Setting for 3–5-year-old children born VPT. Although Active-Prem has been designed to align with the needs and preferences of children born VPT and their families, with potential to be implementable on a larger scale if found effective, there are some limitations to this study. Children with sensory or behavioural difficulties may find it challenging to wear the accelerometer continuously for 7 days, which may impact data collection completion for our primary outcome measure. Another limitation is the complexity of the Active-Prem intervention; completion of all six steps may be delayed or impacted by potential difficulty recruiting coaches in a timely manner while engagement with parent support (step 2) and coach training (step 4) may differ among participants. Furthermore, both the coach and parent programmes are only available in English, which limits the linguistic diversity of the sample. Finally, although we have chosen to compare two active interventions, rather than intervention and control, the Goal Setting group have less interaction with the study, and therefore, may be more likely to disengage. We are trying to mitigate this limitation by providing families with feedback on their child’s PA at two timepoints throughout the study to support engagement from both groups. Strengths of this study include the use of implementability outcome measures designed to inform any adaptations that may be needed to improve future iterations of the intervention and that the intervention builds on substantial previous research,^2–4 22–25^ as described above in each ‘rationale’ section of the intervention description (table 2). Furthermore, Active-Prem includes two components, the Active Start Active Prems parent programme and the Coaching Essentials: Premature Children in Sport coach programme, which were developed through co-design using the Partnership‐focused Principles‐driven Online co‐Design (P‐POD) approach.^24^ Co-design optimises the likelihood that our intervention meets the needs of end-users, involves behaviour change principles and increases the likelihood of successful future implementation and adoption.^27 51^ Additionally, our project is informed by the conceptual framework of implementability, which describes five interrelated concepts influencing perceptions of healthcare interventions and subsequent implementability.^27^ It proposes that if an intervention is not able to be reasonably delivered (feasible),^52^ delivered as intended (fidelity)^47^ and considered appropriate and suitable from the perspectives of those delivering and receiving the intervention (acceptable),^53^ it is unlikely to be adopted and sustained into practice.^27^ Focusing on feasibility, fidelity and acceptability will increase the likelihood that our intervention is responsive to the preferences of the communities that will use it.^53^ Our overall intention is that our theory informed approach will facilitate the translational potential of Active-Prem. Furthermore, embedding Active-Prem within local communities will enhance the sustainability of both the intervention and the health behaviour (PA) of participants, as children will be more likely to continue their chosen activity after the study concludes.

## Supplementary material

10.1136/bmjopen-2026-124614 online supplemental file 1

10.1136/bmjopen-2026-124614 online supplemental file 2

10.1136/bmjopen-2026-124614 online supplemental file 3

10.1136/bmjopen-2026-124614 online supplemental file 4

10.1136/bmjopen-2026-124614 online supplemental file 5
