# Supplementary material for: Enhancing physical activity in early childhood for 3–5-year-old children born very preterm: protocol for an Australian randomised controlled trial of Active-Prem
Source: BMJ Open. 2026 Nov 2;16(11):e124614. doi: 10.1136/bmjopen-2026-124614 (PMC13735262; doi:10.1136/bmjopen-2026-124614)
Supplement: Supplementary file 1 [file bmjopen-16-11-s001.pdf]

Supplementary A: Active-Prem Plain Language and Consent Forms

Plain Language Statement: Families

Introduction

Thank you for your interest in participating in this research project. The following few pages will provide you with further information about the project, so that you can decide if you would like to take part in this research.

Please take the time to read this information carefully. You may ask questions about anything you don't understand or want to know more about.

Your participation is voluntary. If you don't wish to take part, you don't have to. If you begin participating, you can also stop at any time.

What is this research about?

Physical activity during early childhood is important for children's health and development. Children born very preterm, or less than 32 weeks' gestation, are less physically active on average than their term-born peers and can face greater barriers to participating in physical activities in their local communities.

This research aims to compare two approaches to supporting children aged between 3 to 5 years who were born very preterm to be more physically active. The two intervention approaches are; 1) the Active-Prem intervention and 2) Physical activity goal setting with a physiotherapist or occupational therapist (Goal Setting). We do not know which approach is best. This study will compare the two approaches to find out which is more effective, acceptable for families and cost-effective.

What will we be asked to do?

You and your child will be participating in a randomised controlled trial research study. This means you and your child will complete either the Active-Prem intervention or Goal Setting intervention. To try to make sure the groups are the same, each participant is put into a group by chance (random). You and your child have an equal chance of being in either group, but both you and your child will be in the same group. Neither you nor the researchers can choose which group your child is put in. This research study is designed this way so that the researchers can compare the Active-Prem intervention with the Goal Setting intervention in a way that is fair. Comparing intervention approaches is an important way to find out which should be the best-practice approach.

If you indicate that you and your child want to participate in the study, you will be asked to sign the consent form.

Before you find out which group you and your child will be in, we will ask you to complete a few steps.

Your child will be asked to wear an activity monitor on their ankle for 7 days in a row, 24 hours per day. The activity monitor tracks how much physical activity your child completes during the 7 days. We will post the activity monitor to you and provide a reply-paid envelope to return it.

You and your child will attend a goal-setting session with a physiotherapist or occupational therapist. During this session, the research team member will support you to choose some physical activity goals for your child. This will take place over Zoom or by phone at a time convenient for you.

We will ask you to complete some questionnaires about your child and their development, and your own physical activity levels. These can be completed online and will take around 35-45 minutes to complete, or slightly longer if you have twins/triplets. If you like, you can complete these questionnaires during the goal-setting session. This is the longest set of questionnaires you will be asked to complete for the study.

After you complete these steps, your family will be allocated to either the Active-Prem intervention group or the Goal Setting group.

You and your child will be asked to do different things depending on which group you are randomly assigned. You and your child will be in the same group. You will not have the opportunity to try the group you are not allocated to as part of the study.

What if we are randomly allocated to the Active-Prem group?

If you and your child are in the Active-Prem group, you will be asked to complete the following activities.

Choose a physical activity class in your local community with the help of the Active-Prem team. You will be asked to enrol in this physical activity yourself, including providing any relevant health information about your child.

Participate in an introduction session with your child and the coach of your chosen physical activity. The coach will have completed the Active-Prem coach training.

Take your child to one term of your chosen physical activity once per week for 8-10 weeks.

Participate in an online *Active Start Active Prems* program throughout the Active-Prem study. This program begins after you are randomly allocated to the Active-Prem intervention group and finishes after your child has completed one term of your chosen physical activity. The aim of this program is to support you to support your child's participation in physical activity.
The online program includes self-directed content that you can complete at a time that suits you and 1-3 optional Zoom sessions. The program will take around 5-8 hours, depending on which optional content you would like to complete. You will have around 10-15 weeks to complete the program content.

Complete a goal-setting check-in at the end of the term. Complete some online questionnaires during the term, after the term, and 12-months after you joined the study.
How long will everything take to complete?

- Goal-setting check-in = 15-minute phone call
- Questionnaire before starting *Active Start Active Prems* = 5 minutes
- Questionnaire at the end of each class during term = 2 minutes
- Questionnaire after the term of activity = 15 minutes
- Questionnaire 12 months after joining the study = 10 minutes

Ask your child to wear the activity monitor two more times.

- 7 days of wear (24 hours per day) after the term of activity
- 7 days of wear (24 hours per day) 12 months after enrolling in the study.

What if we are randomly allocated to the Goal Setting group?

If you and your child are in the Goal Setting group, you and your child will be asked to complete the following activities.

Around 2 preschool/school terms after you have completed the first goal setting session with a physiotherapist or occupational therapist, we will ask you to complete a goal check-in session. This will be an opportunity to revisit your original goals and get feedback from the physiotherapist or occupational therapist on your child's physical activity. This goal check is session will take place on the phone at a time convenient to you.

Ask your child to wear the activity monitor two more times over the next 18 months.

- 7 days of wear (24 hours per day) after your goal check-in phone call.
- 7 days of wear (24 hours per day) 12 months after enrolling in the study.

Complete some further questionnaires online.
How long will everything take to complete?

- Questionnaires at the same time as the goal check-in phone call= 15 minutes
- Questionnaire 12 months after joining the study= 10 minutes

Other relevant information

We plan to recruit 204 children and their parents to participate in this research study from across Australia. The study will run for approximately 3 years.

If you are allocated to the Active-Prem group, the study will pay the fees for one term of your chosen physical activity. Please note that for some activities, there may be other costs associated with participating in that activity that the study will not pay for, such as sports shoes or dancewear.

The physical activity classes that are part of the Active-Prem program will be taken by coaches who have received the Active-Prem coach training. The classes are continuing classes available in your local community. They are open to the general public. You may continue to attend an activity chosen as part of the Active-Prem study after the study has been completed. This will be at your own cost.

Can my child participate in therapy and/or sports during the study?

Yes. Participation in this study will not influence whether you and your child can access any interventions or therapy. This study will also not influence your child's participation in any other physical activities they may wish to try.

What are the possible benefits?

We cannot guarantee or promise that you or your child will receive any benefits from research. For all families in the study, you will receive two reports outlining your child's physical activity levels; one approximately 2 preschool/school terms after enrolling in the study, and another 12-months after you first joined the study. This information will come from the activity monitor and information included in the questionnaires.

Goal setting is completed with families in both the Active-Prem group and the Goal Setting group. Goal setting is associated with changes in behaviour and may have positive effects on your child's physical activity levels.

For families in both groups, participation in this study may have positive effects on your child's participation in physical activity. For parents in the Active-Prem group, the online parent program may have a positive effect on parents' knowledge and skills to support their child's physical activity.

What are the possible risks?

We do not anticipate any risks or side-effects from participating in this study, other than the risks expected from participation in any physical activity. Risks of participating in physical activity are minimal and can include, for example, muscle soreness or tiredness. Overall, physical activity is recommended as part of a healthy lifestyle.

For all families in the study, being involved requires time from parents to complete online questionnaires and goal-setting sessions. For children in the study, wearing the activity monitor may cause annoyance. For families in the Active-Prem group, being involved in the intervention will involve time commitment to attend classes and complete the parent support program.

Sometimes answering questions about your child's wellbeing or development may be upsetting. If you have become upset or distressed as a result of participation in the research, we can arrange appropriate support. This support may be provided by a qualified member of the research team, or a professional who is not in the team. If you have any concerns or questions related to the study please contact Professor Alicia Spittle Chief Investigator on (03) 9035 5340.

Do we have to take part?

No. Participation is completely voluntary. You do not have to take part if you do not want to. You can also withdraw your child from the study at any time. You just need to let us know. You do not need to tell us why you are taking your child out of the study. If your child leaves the study, we will use the information we have collected about you and your child. If you do not want us to use this information, please tell us. If you do not want us to keep using information about you and your child, we will securely destroy it.

What happens if we're in the Active-Prem group and our coach withdraws from the study?

The coaches involved in this study have also consented to take part. Just like you, they have the option to withdraw from the study at any time. If your coach does decide to withdraw from the study before they have completed the Active-Prem coach training, the study team will make all reasonable effort to find another coach or another activity for your child. This may involve attending the activity in the following term. If a coach withdraws from the study after the term begins, you will still be able to complete the term of activity.

Will we hear about the results of this project?

Yes! We will share the results of the study with you at the conclusion of the project. The results will be summarised in an end-of-study newsletter which you will be provided via email. In addition, we plan on sharing the results of this study with the scientific community, such as through publications or conference presentations. We will also work with our research partners (including Life's Little Treasures Foundation, Miracle Babies Foundation, Gymnastics Victoria, KinderBallet, AllSportz and The Circus Spot) to share our findings with the broader community.

What will happen to information about me and my child?

We will collect and use personal and health information about you and your child for research purposes. By signing the consent form you consent to the research staff collecting and using

personal information about you and your child for the research project. This information we collected will include:

- Personal information, such as your child's date of birth
- Health information, such as your child's gestational age
- Physical activity data
- Your viewpoints and opinions, such as those shared in online questionnaires
- Your participation in the *Active Start Active Prems* online program

Any information obtained in connection with this research project that can identify you or your child will remain confidential. We will store information about you and your child securely in a password protected databases at The University of Melbourne. Only the researchers involved with this study and The University of Melbourne ethics committee can have access to this information. It will be disclosed only with your permission, or as required by law. There are three exceptions for families allocated to the Active-Prem group:

- The Active-Prem team may share your contact details with your matched coach.
- Your email address will be shared with Wix.com so you can login to the *Active Start Active Prems* parent program.
- Your mobile phone number will be shared with a secure, Australian-based email-to-SMS service to enable weekly questionnaires completed during the term of activity to be sent via text (we use an opt-in approach, you may receive these weekly questionnaires via email if you prefer).

More information about these three exceptions is below.

Information about you and your child that is stored at the University of Melbourne will be re-identifiable. This means that we will remove identifying information such as your child's name and give the information a special code number. Only the research team can match your child's name to their code number, if it is necessary to do so.

To advance science, medicine and public health, we may also need to share your child's de-identified data with other ethically approved research studies or medical journals. If we need to do this, we will remove identifying details such as your child's name, date of birth and address and give the data a special code number. Only the research team on this study will be able to match your child's name to their code number.

It is anticipated that the results of this research study will be published and/or presented in a variety of forums. In any publication and/or presentation, information will be provided in such a way that you and your child cannot be identified, except with your permission.

You have the right to access and correct the information we collect and store about your child. This is in line with relevant Australian and/or Victorian privacy and other relevant laws. Please contact us if you would like to access this information.

Information will be stored on a password- protected server and REDCap computer database at the University of Melbourne for 15 years after the youngest child in the study turns 18, after which time it may be destroyed or kept indefinitely. Only the study researchers will have access to the data.

You must be aware that the information collected about your child may at some point not be able to be identified, once the identifying information has been removed. Access to information about you after this point, will not be possible.

If we are in the Active-Prem group, what information will be given to the physical activity provider or coach?

For families in the Active-Prem intervention group, your contact details, including phone number and email address, as well as the names of you and your child, may be shared with the physical activity provider or coach of your chosen activity. The study team will not provide any further information to the coach. It will be your responsibility to provide important information, including relevant details about your child's health and development, when you enrol your child in the term of physical activity.

If we are in the Active-Prem group, what do I need to know about information and privacy when accessing the *Active Start Active Prems* online program?

The *Active Start Active Prems* online parent program is hosted on Wix.com.

Your email address is required to login into the website. This is the only personal information required to access and use the *Active Start Active Prems* online program. The *Active Start Active Prems* program also contains an online discussion forum. This discussion forum is optional. You do not need to participate in the discussion forum if you do not wish to. Only members of the research team and other participants in the Active-Prem group will be able to see any information that you share on the discussion forum, as the website is password protected.

The *Active Start Active Prems* website is hosted and controlled by the independent third party, including the data that is collected at any time when the website is used by you (such as your contributions to the discussion board or which pages you visit). By using the *Active Start Active Prems* website for this Study, you acknowledge and understand that you are agreeing to the terms and conditions of use of the independent third party, which may be found on its website at the following address: https://www.wix.com/about/terms-of-use?referralAdditionalInfo=realDeal

The Wix.com platform is hosted outside of Australia. If you provide consent third-party service provider Wix.com may be used to collect data for the Study. This means that your data (your email and anything you write on the discussion board) is stored outside of the University of Melbourne and will be subject to Wix.com's Terms of Service and Privacy Policy: https://www.wix.com/about/privacy

All other information you provide as part of your participation in the study will be stored securely on University of Melbourne servers.

If we are in the Active-Prem group, what do I need to know about information and privacy when receiving questionnaires via text message?

If you opt in to receive the weekly after class questionnaires via text message, your mobile phone number will be provided to ClickSend, an Australian-based email-to-SMS provider. ClickSend will keep your phone number for 4 months and will not sell your information to any third parties. Any information you input into questionnaires sent via text will only be securely stored on University of Melbourne REDCap and not accessible by ClickSend.

Who is funding this project?

This project is funded by a project grant from the Medical Research Future Fund's Maternal Health and Healthy Lifestyles grant opportunity.

Where can I get further information?

If you would like more information about the project, please contact:

Project Supervisor: Prof Alicia Spittle

Tel: +61 3 90355390

Email: aspittle@unimelb.edu.au

Project coordinator: Dr Kate Cameron

Tel: +61 420 508 595

Email: active-prem@unimelb.edu.au or k.cameron@unimelb.edu.au

Who can I contact if I have any concerns about the project?

This project has human research ethics approval from The University of Melbourne (2025-31093-64376-3). If you have any concerns or complaints about the conduct of this research project, which you do not wish to discuss with the research team, you should contact the Research Integrity Administrator, Office of Research Ethics and Integrity, University of Melbourne, VIC 3010. Tel: +61 3 8344 1376 or Email: research-integrity@unimelb.edu.au. All complaints will be treated confidentially. In any correspondence please provide the name of the research team and/or the name or ethics ID number of the research project.

Consent Form

Participating child's first name: ________________________________

Participating child's last name ________________________________

Participating parent's first name ________________________________

Participating parent's last name ________________________________

1. I consent for my child and I to participate in this project, the details of which have been explained to me, and I have been provided with a written plain language statement to keep.
2. I understand that the purpose of this research is to compare two approaches to supporting children aged between 3 to 5 years who were born very preterm to be more physically active. The two intervention approaches are; 1) the Active-Prem intervention and 2) Physical activity goal setting with a physiotherapist or occupational therapist (Goal Setting).
3. I understand that the participation of my child and I in this project is for research purposes only.
4. I acknowledge that the possible effects of participating in this research project have been explained to my satisfaction.
5. In this project my child and I will be required to complete the activities listed in the plain language statement, relevant to the group my child and I are allocated to.
6. I understand that the participation of my child and I in this project is voluntary and that I am free to withdraw myself and my child from this project anytime without explanation or prejudice and to withdraw any unprocessed data that I have provided.
7. I understand that the data from this research will be stored at the University of Melbourne and may be destroyed 15 years after the youngest participant in the study turn 18.
8. I understand that de-identified data and analysed results from this project may be used for further projects related to improving physical activity participation for children born preterm.
9. I consent to my email address being provided to third-party service provider Wix.com. I acknowledge that information captured will be subject to Wix.com's Terms of Service and Privacy Policy, and information will be stored and processed by Wix.com overseas. I understand that no other personal information will be provided to Wix.com.

10. I understand that by using the *Active Start Active Prems* website I am agreeing to the terms and conditions of use of Wix.com, which may be found on its website at the following address: https://www.wix.com/about/terms-of-use?referralAdditionalInfo=realDeal

11. I understand that my personal information is collected and handled in accordance with the *Privacy and Data Protection Act 2014* (Vic).

12. I understand that after I sign and return this consent form, it will be retained by the researcher.

[] Please check this box if you agree with all of the above statements.

Signature of parent/carer: ______________________ **Date:** ____________________

Plain Language Statement: Coaches

Introduction

Thank you for your interest in participating in this research project. The following few pages will provide you with further information about the project, so that you can decide if you would like to take part in this research.

Please take the time to read this information carefully. You may ask questions about anything you don't understand or want to know more about.

Your participation is voluntary. If you don't wish to take part, you don't have to. If you begin participating, you can also stop at any time.

What is this research about?

Physical activity during the preschool years is important for children's health and development. Children born very preterm, or less than 32 weeks' gestation, are less physically active on average than their term-born peers and can face greater barriers to participating in physical activities in their local communities.

The Active-Prem program is a multi-step intervention aiming to improve community-based physical activity participation for 3–5-year-old children who were born very preterm. Part of this intervention involves partnering with coaches and physical activity providers. Children in the Active-Prem study will be matched with an activity and a coach in their local community. Coaches will complete an online training program called *Coaching Essentials: Premature Children in Sport* and then children will attend one term of physical activity with their matched coach.

The aim of the Active-Prem study is to trial the Active-Prem intervention to understand if it is effective and acceptable for both coaches and families, and if it is cost-effective to run.

What will I be asked to do?

Should you agree to participate you, you will be asked to:

- Complete the *Coaching Essentials: Premature Children in Sport* program
- Facilitate the enrolment of one or more participating child or children in your class
- Complete an introductory session with the participating child or children and their parent(s) or carer(s).
- Include one or more participating child or children in your class for one term
- Use the tools and strategies included in the *Coaching Essentials* program in during your class.
- Maintain the confidentiality of the child or children enrolled in your class
- Complete some online questionnaires throughout the program

Your participation in Active-Prem will last around 6 months, or two school terms. Further details about what you will be asked to do are described below.

Online training program

You will be asked to complete an online training program called *Coaching Essentials: Premature Children in Sport*. This program is delivered online on a platform called FutureLearn. It will take you around 8 hours to complete the online training. You will have approximately four weeks to complete the program. There will also be optional Coach Support Workshops will which take place on Zoom. The online program includes one assessment task that you will need to complete as part of the *Coaching Essentials* program. There will be support to complete this task if you need.

Enrolling a participating child into your class

Once a child (or children) has been matched to your class, we ask that you enrol them as you would any other child. It is the responsibility of you and the physical activity organisation you work for to obtain relevant information, such as emergency contacts and medical conditions, from the family. To maintain confidentiality, we require that you do not discuss any participating child's involvement in the Active-Prem study or any child's personal details gained through the enrolment process, except with the owner of the physical activity organisation (if this is not you), members of the research team or the child's family.

Introductory session

Before the matched child or children's first class, you will be asked to complete an introductory session with the children and their parent(s) or carer(s). There is more detailed information about what this introductory session involves and how to prepare in the *Coaching Essentials* program. The introductory session should take place at a time that suits you and the participating family. It will be approximately 60 minutes long and include approximately 30 minutes of 'getting to know you' time and approximately 30 minutes of tailored introduction to foundational skills of activity.

Coaching your class

Once the participating child or children have started class, you will be asked to incorporate the strategies discussed in the *Coaching Essentials* program into your class. These strategies should not affect the overall content or aims of your class. More detailed information on this topic will be provided during the *Coaching Essentials* program. We do not require that you prioritise the participating children in any way over non-participating children while coaching your class. The participating children will be enrolled for one term of the activity.

Questionnaires

As this is a research project, there are a few questionnaires we ask you to complete.

At the start of Active-Prem

We will ask you to complete a questionnaire about your thoughts on the Active-Prem program and about including children born preterm in your class. These questionnaires will be completed online (phone or computer) and will take around 15-20 minutes.

During the term of activity

After each class the participating child is enrolled in, we ask that you complete a brief 2-minute questionnaire (2 questions) about the child's attendance and involvement in the class. A link to

this questionnaire will be sent as a text message on the day of the class. Twice throughout the term, this questionnaire will have a few additional few questions (5-10 minutes).

At the end of the term

We will ask you to complete the same questionnaire you completed at the start of the project. These questionnaires will be completed online (phone or computer) and will take around 15-20 minutes.

Other relevant information

We plan to recruit 204 children and their parents to participate in this research study. The study will run for approximately 3 years.

Parents of children participating in the Active-Prem program will also complete an online program. This program aims to support parents to better support their child's participation in community-based physical activity.

You will be gifted a $200 voucher as a thank you for participating in the program and an additional $50 voucher when the final questionnaires are completed at the end of the term.

What are the possible benefits?

You will have the opportunity to complete a co-designed online professional development program, *Coaching Essentials: Premature Children in Sport,* as part of your participation in this study. You will receive a certificate of completion for *Coaching Essentials*. We hope the content of this program will add to your coaching toolbox and be applicable for other children and classes. In addition, you will have at least one additional child enrolled in your class. Finally, in participating in a research study, you will provide a valuable contribution to research that is supporting children born very preterm to thrive.

What are the possible risks?

We do not anticipate any risks from participating in this study. There is a small chance that you may find some of the information included in the *Coaching Essentials* program upsetting. If you have become upset or distressed as a result of participation in the research, we can arrange appropriate support. This support may be provided by a qualified member of the research team, or a professional who is not in the team. If you have any concerns or questions related to the study please contact Professor Alicia Spittle Chief Investigator on (03) 9035 5340.

Do I have to take part?

No. Participation is completely voluntary. You are able to withdraw at any time. You just need to tell us that you would like to withdraw. You do not need to tell us why. If you withdraw from the study, we will use the information we have collected from you. If you do not want us to use this information, please tell us.

What happens if the child in my class withdraws from the study?

The children and parents in this study have also consented to take part. Just like you they have the option to withdraw at any time. If the child attending your class does withdraw, the research team will let you know. You won't need to complete all of the questionnaires during the term after they withdraw. If the child in your class withdraws, we still encourage you to remain participating in the study if you want to.

Will I hear about the results of this project?

Yes! We will share the results of the study with you at the conclusion of the project. The results will be summarised in an end-of-study newsletter which you will be provided via email. In addition, we plan on sharing the results of this study with the scientific community, such as through publications or conference presentations. We will also work with our research partners (including Life's Little Treasures Foundation, Miracle Babies Foundation, Gymnastics Victoria, KinderBallet, AllSportz, Kelly Mini Sports and The Circus Spot) to share our findings with the broader community.

What will happen to information about me?

We will collect and use personal information about you for research purposes. By signing the consent form you consent to the research staff collecting and using personal information about you for the research project.

Any information obtained in connection with this research project that can identify you will remain confidential. It will be disclosed only with your permission, or as required by law. By consenting you are providing permission for the research team to share some information with families allocated to your class. This is described below.

We will store your information securely in the Department of Physiotherapy at The University of Melbourne. Only the researchers involved with this study and The University of Melbourne ethics committee can have access to this information. We can disclose the information only with your permission except as required by law.

The stored information will be re-identifiable. This means that we will remove identifying information such as your name and give the information a special code number. Only the research team can match your name to their code number, if it is necessary to do so.

If you opt in to receive the weekly after class questionnaires via text message, your mobile phone number will be provided to ClickSend, an Australian-based email-to-SMS provider. ClickSend will keep your phone number for 4 months and will not sell your information to any third parties. Any information you input into questionnaires sent via text will only be securely stored on University of Melbourne REDCap and not accessible by ClickSend.

To advance science, medicine and public health, we may also need to share your de-identified data with other ethically approved research studies or medical journals. If we need to do this, we will remove identifying details such as your name and give the data a special code number. Only the research team on this study will be able to match your name to the code number.

It is anticipated that the results of this research study will be published and or presented in a variety of forums. In any publication and/or presentation, information will be provided in such a way that you cannot be identified, except with your permission.

You have the right to access and correct the information we collect and store about you. This is in line with relevant Australian and/ or Victorian privacy and other relevant laws. Please contact us if you would like to access this information.

All information will be electronic information and will be stored on a password- protected server and REDCap computer database at the University of Melbourne for 15 years after the youngest child in the study turns 18, after which time it may be destroyed or kept indefinitely. Only the study researchers will have access to the data.

You must be aware that the information collected about you may at some point not be able to be identified once the identifying information has been removed. Access to information about you after this point will not be possible.

What information will be shared with families?

To facilitate the enrolment of children participating in Active-Prem into your physical activity class, families may be provided with your contact details, including your name, phone number or email address. Wherever possible, the research team will only share information that is already publicly available (e.g. the contact email address for your physical activity organisation). The research team will let you know which information they share with families.

What do I need to know about the privacy and confidentiality of families participating in the study?

As a coach participating in this study, you will have access to personal and health information about any children participating in Active-Prem who are allocated to your class. By consenting to participate in this study, you are agreeing to maintaining the privacy and confidentiality of any children or parents enrolled in this study. This is outlined in a Letter of Agreement (attached) which your physical activity organisation will sign prior to any participating child attending a class.

Who is funding this project?

This project is funded by a project grant from the Medical Research Future Fund's Maternal Health and Healthy Lifestyles grant opportunity.

Where can I get further information?

If you would like more information about the project, please contact:

Project Supervisor: Prof Alicia Spittle

Tel: +61 3 90355390

Email: aspittle@unimelb.edu.au

Project coordinator: Dr Kate Cameron

Tel: +61 420 508 595
Email: active-prem@unimelb.edu.au or k.cameron@unimelb.edu.au

Who can I contact if I have any concerns about the project?

This project has human research ethics approval from The University of Melbourne, project ID 31093. If you have any concerns or complaints about the conduct of this research project, which you do not wish to discuss with the research team, you should contact the Research Integrity Administrator, Office of Research Ethics and Integrity, University of Melbourne, VIC 3010. Tel: +61 3 8344 1376 or Email: research-integrity@unimelb.edu.au. All complaints will be treated confidentially. In any correspondence please provide the name of the research team and/or the name or ethics ID number of the research project.

Consent Form

Name of Participant: ______________________________

13. I consent to participate in this project, the details of which have been explained to me, and I have been provided with a written plain language statement to keep.

14. I understand that the purpose of this research is to investigate the effectiveness of the Active-Prem intervention.

15. I understand that my participation in this project is for research purposes only.

16. I acknowledge that the possible effects of participating in this research project have been explained to my satisfaction.

17. In this project I will be required to:
    - Complete the *Coaching Essentials: Premature Children in Sport* program
    - Facilitate the enrolment of one or more participating child in my class
    - Complete an introductory session with the participating child or children and their parent(s) or carer(s).
    - Include one or more participating children in my class for one term
    - Use the tools and strategies included in the *Coaching Essentials* program in during my class.
    - Maintain the confidentiality of the child or children enrolled in my class
    - Complete some online questionnaires throughout the program

18. I understand that my participation is voluntary and that I am free to withdraw from this project anytime without explanation or prejudice and to withdraw any unprocessed data that I have provided.

19. I understand that the data from this research will be stored at the University of Melbourne and may be destroyed 15 years after the youngest participant in the study turn 18.

20. I have been informed that the confidentiality of the information I provide will be safeguarded subject to any legal requirements; my data will be password protected and accessible only by the named researchers.

21. I understand that by consenting to this project, I am agreeing to maintain the privacy and confidentiality of families enrolled in Active-Prem.

22. I understand that after I sign and return this consent form, it will be retained by the researcher.

Participant Signature: ______________________ **Date:** ______________
