# Supplementary material for: Enhancing physical activity in early childhood for 3–5-year-old children born very preterm: protocol for an Australian randomised controlled trial of Active-Prem
Source: BMJ Open. 2026 Nov 2;16(11):e124614. doi: 10.1136/bmjopen-2026-124614 (PMC13735262; doi:10.1136/bmjopen-2026-124614)
Supplement: Supplementary file 2 [file bmjopen-16-11-s002.pdf]

Supplementary B: Trial oversight, safety and data management

Name and contact information for the trial sponsor

Trial sponsor: University of Melbourne.

Contact: Professor Alicia Spittle: aspittle@unimelb.edu.au

Trial Oversight

The Active-Prem steering committee, which consists of the study CI (AS), the study coordinator (KC) and key study investigators, will maintain oversight over the complete Active-Prem project, ensuring it is conducted to the required standards. The Active-Prem Steering Committee will meet regularly, meetings will be documented, and attendees will be listed.

Active-Prem working group 2

The Active-Prem working group 2 is a group of Active-Prem investigators with expertise in running clinical trials, other relevant research expertise (e.g. health economics), or lived experience of preterm birth. This group is responsible for supporting the trial management group with conduct and progress of the trial, and to facilitate smooth conduct of the trial.

Trial management group

The trial management group will consist of a small group of people who will be responsible for the day-to-day management of the trial. The group will include the study CI and study coordinator, as well as relevant research staff responsible for day-to-day running of the trial. The group will closely review all aspects of the conduct and progress of the trial, ensuring that there is a forum for identifying and addressing issues. Meeting minutes will be taken with attendees listed, pertinent emails retained, and phone calls documented.

Safety Monitoring

Safety oversight will be under the direction of a Data and Safety Monitoring Board (DSMB). It is composed of individuals with the appropriate expertise, including at least three independent clinicians and/or biostatisticians who, collectively, have experience in the management of paediatrics, biostatistics and the conduct and monitoring of randomised controlled trials. Members of the DSMB will be independent of trial conduct. The DSMB will meet at least annually evaluate the accumulating study data on safety, study conduct and progress. The DMSB will operate under the rules of an approved charter that was written and reviewed at the organisational meeting of the DSMB. At this time, each data element that the DSMB needs to assess was clearly defined. The DSMB will provide its input to the Sponsor-Investigator.

The safety data that are being collected as part of this trial and that will be included in the DSMB report are; adverse events, related adverse events, serious adverse events, unexpected, related serious adverse events, significant safety issue and urgent safety measures. The reporting of safety events for this study follows the Australian National Health and Medical

Research Council's 'Safety monitoring and reporting in clinical trials involving therapeutic goods' (November 2016).

DSMB will provide its input to the Sponsor-Investigator.

Risk assessment

Overall, Active-Prem is categorised as a low-risk trial. Risks have been categorised according to the Melbourne Clinical Trials Centre 'CRDO RISK ASSESSMENT AND RISK MANAGEMENT TOOL'

For parents, there is an unlikely possibility of emotional distress (classified as minor possible harm) including their child in a mainstream physical activity. The Active-Prem intervention is designed to support parents through this process. If parents require additional psychological support for any distress, they will be provided with referral/information about appropriate support services with their permission following a debriefing phone call with a study investigator.

For parents and coaches, there is an unlikely possibility of emotional discomfort (classified as minor possible harm) experienced due to the content of the co-designed online programs, *Coaching Essentials: Premature Children in Sport* and *Active Start Active Prems* parent program. This risk has been mitigated by the comprehensive co-design process, which involved parents and coaches at all stages of the design and development. This process enhances the likelihood that content will be delivered in a way that is acceptable and sensitive to parents and coaches. Within the parent program, this risk has been further mitigated by making topics that may be uncomfortable (e.g. discussions about parent concerns about child not fitting in) optional content. Furthermore, both the parent program *Coaching Essentials* will contain information about where to seek further support if any topic results in feelings of discomfort or distress.

For children, there is an unlikely possibility of moderate harm (e.g. musculoskeletal injury) from participating in physical activity classes. We have considered this an accepted risk, as this is the same level risk (low risk) as experienced by any child participating in physical activity within their local community, whether or not this is in a trial context. The overall benefits of physical activity (outlined in the introduction) are greater than the low risk of harm. Families will be made aware of this low risk in the plain language statement (Supplementary A) provided prior to consent. Any incidents of adverse events will be recorded and monitored as detailed below.

Definitions

<u>Participant-specific adverse events</u>

<u>Adverse Event (AE)</u>: Any untoward medical occurrence in a clinical trial participant receiving an intervention and does not necessarily have a causal relationship with this intervention. Information on Non-serious adverse Events will **only** be collected if they occur during trial activities (such as attending classes) or they are related to the trial in some way (e.g. reason for withdrawing from trial).

<u>Related Adverse Event*</u>: An adverse event that is judged as having a reasonable causal relationship with the trial intervention.

Note: The expression ‘reasonable causal relationship’ means to convey, in general, that there is evidence or argument to suggest a causal relationship.

Serious Adverse Event (SAE): Any adverse event/adverse reaction that results in death, is life threatening, requires hospitalisation or prolongation of existing hospitalisation, results in persistent or significant disability or incapacity or is a congenital anomaly or birth defect.

Note: Life-threatening refers to an event in which the participant was at risk of death at the time of the event. It does not refer to an event that hypothetically might have caused death if it were more severe.

Medical and scientific judgement should be exercised in deciding whether an adverse event should be classified as serious in other situations. Important medical events that are not immediately life-threatening or do not result in death or hospitalisation but may jeopardise the participant or may require intervention to prevent one of the other outcomes listed in this definition should also be considered serious.

Unexpected and Related Serious Adverse Event (URSAE)*: An adverse event that is:

- Serious – meets the definition of an SAE (see above)
- Related – resulted from administration of the trial intervention
- Unexpected – the event is not described in the protocol as an expected occurrence

Safety issues (requiring expedited reporting)

The following definitions describe additional safety events that require expedited reporting to stakeholders including the Sponsor, Investigators, HREC, and local governance office.

Significant Safety Issue (SSI): A safety issue that could adversely affect the safety of participants or materially impact on the continued ethical acceptability or conduct of the trial. Comment: An SSI is a new safety issue or validated signal considered by the Sponsor in relation to the intervention that requires urgent attention of stakeholders. This may be because of the seriousness and potential impact on the benefit-risk balance of the intervention, which could prompt regulatory action and/or changes to the overall conduct of the clinical trial, including the monitoring of safety and/or the administration of the intervention.

Urgent Safety Measure (USM): A measure required to be taken in order to eliminate an immediate hazard to a participant’s health or safety. Note: This is a type of SSI that can be instigated by either the investigator and can be implemented before seeking approval from HRECs or institutions.

Capturing and eliciting adverse event/reaction information

Adverse events (non-serious and serious) that are potentially related to the intervention (e.g. occur during a physical activity class OR during engagement with the online training materials) or will impact ongoing participation in the trial, will be captured from the time of enrolment until conclusion of the intervention. Participating families and coaches will be asked to alert the research team of any relevant adverse events.

Documentation of Adverse Events

For the purposes of this trial, Adverse Events will be recorded if they are possibly, probably or definitely related to the intervention (or may be partially related to the intervention) or will impact the ongoing participation of the participant in the intervention (i.e. the participant may

discontinue classes if they become unwell). Conditions that are present during screening, and do not deteriorate, will not be considered adverse events.

The AE will be described in an Expedited Safety Form (Appendix V), and include:

- The likelihood of the relationship of the AE to the intervention (Unrelated, Possible, Probable, Definite)

The following if the adverse event is related to the intervention:

- A description of the Adverse Event
- The onset date, duration, date of resolution
- Severity (mild, moderate or severe)
- Seriousness
- Any action taken by the study team
- The outcome

For AEs related to the intervention: AEs will be followed to adequate resolution, where possible, with changes in severity noted.
If an Adverse Event is unrelated to the intervention AND impacts on the participant being able to complete the intervention (e.g. a participant becomes unwell and can no longer attend classes), minimal details will be collected by the research team (as this information is health information not required for the study outcomes), and the participant will be noted as *discontinued from the intervention for medical reasons not related to the intervention*.
Note: Young children often miss classes due to colds and other mild infections. These will be noted as non-attendance of class due to illness, but will not be considered AEs as this is not an untoward medical event.

Assessing the relatedness (causality) of a participant's AE

All adverse events must have their relationship to the trial intervention assessed by the investigator who evaluates the adverse event based on temporal relationship and his/her clinical judgment. The degree of certainty about causality will be graded using the categories below.

The relationship of the event to the trial intervention will be assessed as follows:

- **Unrelated:** There is no association between the trial intervention and the reported event. AEs in this category do not have a reasonable temporal relationship to exposure to the intervention, or can be explained by a commonly occurring alternative aetiology.
- **Possible:** The event could have cause or contributed to the AE. AEs in this category follow a reasonable temporal sequence from the time of exposure to the intervention and/or follow a known response pattern to the intervention, but could also have been produced by other factors.
- **Probable:** The association of the event with the trial intervention seems likely. AEs in this category follow a reasonable temporal sequence from the time of exposure to the intervention and are consistent with the known action of the intervention, known or previously reported adverse events related the intervention, or judgement based on the investigator's clinical experience.
- **Definite:** The AE is a consequence of administration of the trial intervention. AEs in this category cannot be explained by progression of the condition, concurrent illness or medication, or other factors. Such events may be widely documented as having an association with the intervention or that they occur after rechallenge.

Assessing the seriousness of a participant's AE

The seriousness of an AE will be assessed by an investigator according to the definition in in the preceding section on definitions with the following exception(s):

- AEs unrelated to the trial

Assessing the expectedness and severity of a participant's AE

Only AEs related to the intervention will be assessed for expectedness and severity. The investigator will be responsible for determining whether an AE is expected or unexpected. An AE will be considered unexpected if the nature, severity, or frequency of the event is not consistent with the risk information previously described for the trial intervention.

The severity of an Adverse Event will be assessed:

- The severity of an Adverse Event will be assessed as follows:
  - o **Mild**: Events that require minimal or no treatment and do not interfere with the participant's daily activities.
  - o **Moderate**: Events that cause sufficient discomfort to interfere with daily activity and/or require a simple dose of medication.
  - o **Severe:** Events that prevent usual daily activity or require complex treatment.

Reporting of safety events

Principal Investigator Reporting Procedures

The Principal Investigator/delegate is responsible for recording all safety events in the source document.

The Site Principal Investigator is responsible for expedited reporting (within 24 hours of becoming aware of the event) to the Sponsor-Investigator the following safety events:

1. Urgent Safety Measures
2. Unexpected and Related Serious Adverse Events
3. All Serious Adverse Events related to the intervention.

Urgent Safety Measures, Unexpected and Related Serious Adverse Events and Serious Adverse Events should be submitted to the Sponsor-Investigator as soon as possible (but within 24 hours of the first knowledge of the event) using the trial Expedited Safety Report Form (see Appendix V).

The Site Principal Investigator is also responsible for reporting Significant Safety Issues, Urgent Safety Measures and URSAEs to their research governance office within 72 hours of becoming aware of the event and in accordance with their local governance authorization.

The Principal Investigator/Sponsor is responsible for the following reporting to the HREC(s):

1. All Significant Safety Issues that meet the definition of a USM within 72 hours of becoming aware of the issue.
2. All other Significant Safety Issues within 15 calendar days of instigating or becoming aware of the issue
3. For Significant Safety Issues leading to an amendment of trial documentation:
   a. Submit details of the Significant Safety Issue without undue delay and no later than 15 calendar days of becoming aware of the issue.

   b. Submit amendment to the HREC without undue delay.
4. For <u>Significant Safety Issues</u> leading to temporary halt or early termination of a trial for safety reasons:
   a. Communicate reasons, scope of halt, measures taken, further actions planned without undue delay and no later than 15 calendar days of decision to halt.
   b. For a temporary halt, notify the Site Principal Investigators and approving HRECs when the trial restarts, including evidence that it is safe to do so.

The Sponsor is responsible for providing the additional safety information to the approving HREC:

1. Provide an annual safety report, including a summary of the evolving safety profile of the trial

The Sponsor is also responsible for providing any updated safety information to all Site Principal Investigators.

Amendments to the protocol

This trial will be conducted in compliance with the current version of the protocol. Any change to the protocol document or Informed Consent Form that affects the scientific intent, trial design, participant safety, or may affect a participant's willingness to continue participation in the trial will be considered an amendment, and therefore will be written and filed as an amendment to this protocol and/or informed consent form. All such amendments will be submitted to the HREC, for approval prior to being implemented.

DATA AND INFORMATION MANAGEMENT

Overview

The Principal Investigator is responsible for storing essential trial documents relevant to data management and maintaining a record of the location of the data management-related Essential Documents.

The Principal Investigator is responsible for maintaining adequate and accurate source documents that include all key observations on all participants. Source data will be attributable, legible (including any changes or corrections), contemporaneous, original, accurate, complete, consistent, enduring and available. Changes to source data (hardcopy and electronic) must be traceable, must not obscure the original entry, and must be explained where this is necessary.

The Principal Investigator will also be responsible for ensuring that the collected and reported data is accurate, legible, complete, entered in a timely manner and enduring. To maintain the integrity of the data, any changes to data (hardcopy and electronic) must be traceable, must not obscure the original entry, and must be explained where this is necessary.

Any person delegated to collect data, perform data entry or sign for data completeness will be recorded on the delegation log and will be trained to perform these trial-related duties and functions.

Data management

Data generation

In this trial, the following types of data will be collected:

- personal identifying information (e.g. names, dates of birth, contact details)
- sensitive information including health data (gestational ages, medical history, developmental questionnaires)
- physical activity data (using accelerometers)
- participant viewpoints and perspectives
- participant engagement in online programs (*Coaching essentials* and *Active Start Active Prems* parent program.
- aspects of program feasibility, including # families and coaches expressing interest in the study, # participants, attendance at the physical activity, #withdrawing.
- interview recordings and transcripts for planned semi-structured interviews

Data capture methods and data use, storage, access and disclosure during the trial

<u>Data collection methods</u>

Data for this trial will be collected using electronic data collection forms (in REDCap) which will be completed either directly by the participant (parent or coach) or by a member of the research team.

Physical activity data will be collected using tri-axial accelerometry.

<u>Use of the data</u>

The data will be used for the analyses specified in the protocol and Statistical Analysis Plan.

Following the completion and analysis of the trial, the data will be retained long-term following the mandatory archive period for use in future research projects.

<u>Storage and access</u>

Hard copy data, including back-up accelerometry data stored on a password protected external harddrive, will be stored in a locked cabinet in a secure location at the University of Melbourne, accessible to the research team only.

Electronic data (including audio) will be securely stored in University of Melbourne's REDCap database system and in files stored on the University of Melbourne Sharepoint, which are backed up nightly. **Files containing private or confidential data will be stored only in locations accessible only by appropriate designated members of the research team.**

REDCap maintains an audit trail of data create/update/delete events that is accessible to project users who are granted permission to view it. Access to REDCap will be provided via an UoM user account. The permissions granted to each user within each REDCap project will be controlled by, and will be the responsibility of, the trial team delegated this task by the Principal Investigator. REDCap has functionality that makes adding and removing users and managing user permissions straightforward. All data transmissions between users and the REDCap server are encrypted. The instructions for data entry to REDCap must be read and the training log signed prior to personnel commencing data entry on REDCap.

Authorised representatives of the sponsoring institution as well as representatives from the HREC, Research Governance Office and regulatory agencies may inspect all documents and records required to be maintained by the Investigator for the participants in this trial.

Disclosure

The trial protocol, documentation, data and all other information generated as part of the trial will be held in strict confidence. No information concerning the trial or the data will be released to any unauthorised third party, without prior written approval of the sponsoring institution. Clinical information will not be released without written permission of the participant, except as necessary for monitoring by the HREC, Research Governance Office or regulatory agencies.

Accelerometer data management

Accelerometers will be explained to families via phone call or teleconferencing. An information handout will be provided in digital (via email) and paper (sent in the post with the accelerometer) formats. Accelerometer non-wear periods will be defined as intervals with ≥20 consecutive minutes of no accelerations or angle changes during waking hours, identified visually and then confirmed using an automated non-wear algorithm. Days with <10 hours of accelerometer wear during waking hours will be classified as poor compliance and excluded from the analysis. Participants with less than 3 days of acceptable accelerometer data will be treated as missing data. Accelerometer-measured outcomes account for the spectrum of movement behaviours over 24-hours and include: 1) total PA (minutes/day) determined by summing each non-stationary minute during waking hours; 2) time (minutes) spent stepping per day (stepping PA, primary outcome); 3) total number of steps per day; 4) moderate-vigorous PA, determined by summing the time spent in fast steps per day (minutes); and 5) stationary time (minutes/day) obtained by summing each minute with no recorded steps during waking hours.[51]

Data confidentiality

Data confidentiality

Participant confidentiality is strictly held in trust by the Principal Investigator, participating investigators, research staff, and the sponsoring institution and their agents.

To preserve confidentiality and reduce the risk of identification during collection, analysis and storage of data and information, the following will be undertaken:

(1) The number of private/confidential variables collected for each individual has been minimised. The data collected will be limited to that required to address the primary and secondary objectives, as well as to conduct the trial (e.g. families will need to provide postal addresses to send out accelerometers)

(2) Participant data will be identified through use of a unique participant trial number assigned to the trial participant ("re-identifiable"). The Principal Investigator is responsible for the storage of a master-file of names and other identifiable data with the participant ID; access to this document will only be available to members of the research team who will need to identify participants to collect further data or undertake study assessments. The master file will take the form of a REDCap database, while a separate REDCap database will be used to collect and store study outcome measures and other non-identifiable sensitive or health data.

Quality assurance

All questionnaire data entered directly into REDCap by participants will be screened for completeness by a research team member, and participants invited to complete any questions they might have missed.
Data entered directly by researchers will be scanned for completeness and accuracy.
Data field limits will be used in all REDCap databases to reduce the likelihood of data entry errors.
Accelerometery data will be downloaded using the AX3 OMGUI Configuration and Analysis Tool (an open-source software) as soon as the accelerometer is returned. At this initial download stage, data will be checked for completeness. In the event that the accelerometer has malfunctioned (i.e., stopped recording during the wear period), families will be contacted and asked whether they would be willing to complete a second trial (optional). Accelerometer data will be processed using custom software developed by A/Prof Ross Clark. A comprehensive guideline will be created to support accelerometer data processing. Processing will be led by one staff member, with difficult datasets to be referred to a senior member of the CI team for second opinion. Accelerometer data will be screened for accuracy to identify errors which may occur due to software or human error.
A Data Safety Management Board (DSMB) will be established and meet at least annually to review and evaluate the accumulating study data on safety, study conduct and progress and will report to the Active-Prem steering committee (see section 10).

Data analysis will be conducted by the study investigators and overseen by the study biostatisticians and Principal Investigator according to the Statistical Analysis Plan, which will be finalised prior to database lock.

Data sharing

Data sharing

Beginning '48' months following analysis and article publication, the following may be made available for use by future researchers from a recognised research institution whose proposed use of the data has been ethically reviewed and approved by the Active-Prem chief investigator and an independent committee and who accept The University of Melbourne's conditions for access:

- Individual participant data that underlie the results reported in this article after de-identification (text, tables, figures and appendices), and that may not identify an individual (even when de-identified).

After the archive period, the data will be anonymised for preservation to reduce the risk of re-identification. The Coordinating Principal Investigator will be responsible for the long-term custodianship of the data.
