# Supplementary material for: Enhancing physical activity in early childhood for 3–5-year-old children born very preterm: protocol for an Australian randomised controlled trial of Active-Prem
Source: BMJ Open. 2026 Nov 2;16(11):e124614. doi: 10.1136/bmjopen-2026-124614 (PMC13735262; doi:10.1136/bmjopen-2026-124614)
Supplement: Supplementary file 3 [file bmjopen-16-11-s003.pdf]

Supplementary C: Active-Prem schedule of assessments and events

	**TRIAL PERIOD**								
	**Enrolment**	**Baseline**	**Intervention period**					**Post intervention**	
**TIME POINT**	Enrolment and consent	Baseline	After group allocation	>3 week before term of activity	>2 weeks before term of activity	Prior to term of activity	Term of activity: approx 8 to 11 weeks	Post intervention	12 months post-randomisation
**Enrolment:**									
**Eligibility screen**	X (family)			X (coach)					
**Informed consent**	X (family)			X (coach)					
**Intervention step:** Completed by Active-Prem group & coaches only									
**1) Goal setting[b]**		X							
**2) Parent support**			Throughout intervention period						
**3) Match with activity**				X					
**4) Coach training**				X	X				
**5) Introduction session**						X			
**6) Term of physical activity**							X		
**Baseline and effectiveness measures:** Completed by all families									
**Accelerometry (7 days)**		X						X	X
**COPM[a] (goal setting)[b]**		X						X	
**Little DCDQ/DCDQ-'07[c]**		X							
**MBQ-C[d] + extra questions**		X						X	X
**Barriers/facilitators (open ended questions)**		X						X	

**YC-PEM[e] (community section)**		X							
**Demographic questionnaire**		X							
**SDQ[f]**		X						X	X
**IPAQ[g]**		X						X	X
**Demographic and effectiveness measures:** Completed by coaches									
**Demographics questionnaire**				X					
**DIBQ[h]**				X				X	
**Implementability measures:** Completed by Active-Prem group OR coaches OR by member of study team.									
**TFA[i] survey**			X family	X coach				X	
**Attendance + Smiley face scale**							X family		
**Attendance + Involvement scale**							X coach		
**Fidelity and adaptability survey: Active-Prem classes**							X coach		
**Fidelity: coach program**					X study team				
**Fidelity: parent program**							X study team		
**File audit (feasibility measures)**	Throughout								
**Economic evaluation measures:** completed by all families.									

**CHU9D**[j]		X						X	X<br>+ additional health care utilisation questions

[a]Canadian Occupational Performance Measure; [b]Goal setting using the COPM forms step 1 of Active-Prem, the control intervention (Goal Setting) AND is used as an effectiveness outcome measure. It is therefore completed by both the Active-Prem and Goal Setting groups; [c]Developmental Coordination Disorder Questionnaire; Little Developmental Coordination Disorder Questionnaire; [d]Movement Behavior Questionnaire; [e]Young Children's Participation and Environment Measure; [f]Strengths and Difficulties Questionnaire; [g]International Physical Activity Questionnaire; [h]Determinants of Implementation Behavior Questionnaire; [i]Theoretical Framework of Acceptability; [j]Child Health Utility instrument.
