# Supplementary material for: Enhancing physical activity in early childhood for 3–5-year-old children born very preterm: protocol for an Australian randomised controlled trial of Active-Prem
Source: BMJ Open. 2026 Nov 2;16(11):e124614. doi: 10.1136/bmjopen-2026-124614 (PMC13735262; doi:10.1136/bmjopen-2026-124614)
Supplement: Supplementary file 4 [file bmjopen-16-11-s004.pdf]

# Supplementary D: Parent-child outcome measures developed specifically for the study.

## Baseline demographic survey

**About [child's name]**

1. What is your relationship with [child's name]?
2. How old is [child's name] today?
3. What date was [child's name] born?
4. What was [child's name]'s due date or EDD?
5. What gestational age was [child's name] born at?
6. Does [child's name] have a diagnosis of cerebral palsy (CP)? [1, Yes | 0, No | 2, seeking diagnosis]
   (if yes or seeking diagnosis)
   a. CP subtype [0, monoplegia | 1, diplegia | 2, hemiplegia | 4, quadriplegia | 5, unsure]
      i. [if hemiplegia chosen] CP side affected [0, right | 1, left]
   b. CP GMFCS level [1, Level I | 2, Level II | 3, Level III | 4, Level IV | 5, Level V | 6, Unsure]
   c. Who diagnosed/is involved with diagnosing [child's name] with CP? (please check multiple options if appropriate) [0, Paediatrician | 1, GP | 2, Physiotherapist | 3, Occupational therapist | 4, can't recall/unsure | 5, Other (please specify)]
   d. At what age was [child's name] diagnosed with CP? [0, less than 1 year | 1, 1 year old | 2, 2 years old | 3, 3 years old | 4, 4 years old | 5, 5 years old | 6 can't recall/unsure]
7. Does [child's name] have a diagnosis of developmental coordination disorder (DCD)? [1, Yes | 0, No | 2, seeking diagnosis]
   (if yes or seeking diagnosis)
   a. Who diagnosed/is involved with diagnosing [child's name] with DCD? Please check multiple if appropriate [0, Paediatrician | 1, Physiotherapist | 2, Occupational therapist | 3, GP or other medical doctor | 4, can't recall/don't know | 5, Other (please specify)]
   b. At what age was [child's name] diagnosed with DCD? [number]
8. Does [child's name] have a diagnosis of autism spectrum disorder (ASD)? [1, Yes | 0, No | 2, seeking diagnosis]
   (if yes or seeking diagnosis)
   a. What ASD diagnosis was given? [0, ASD (DSM V) | 1, ASD level 1 (DSM V) | 2, ASD level 2 (DSM V) | 3, Aspergers (DSM IV) | 4, PDD-NOS (DSM IV) | 5, Autistic disorder (DSM IV) | 6, High functioning autism (DSM IV) | 7, Low functioning autism (DSM IV) | 8, unsure]
   b. Who diagnosed/ is involved with diagnosing [child's name] with ASD? Please check multiple if appropriate [0, Paediatrician | 1, Specialist ASD team | 2, Psychologist | 3, Psychiatrist | 4, GP | 5, Occupational therapist | 6, can't recall | 7, Speech pathologist | 8, Other (please specify)]

   c. At what age was [child's name] diagnosed with ASD? [0, less than 1 year | 1, 1 year old | 2, 2 years old | 3, 3 years old | 4, 4 years old | 5, 5 years old | 6, can't recall/unsure]

9. Does [child's name] have a diagnosis of attention deficit hyperactivity disorder (ADHD) or attention deficit disorder (ADD)? [1, Yes (ADD) | 2, Yes (ADHD) | 0, No | 3, seeking diagnosis]
   (if yes or seeking diagnosis)
   a. Who diagnosed/is involved with diagnosing [child's name] with ADHD or ADD? Please check multiple if appropriate [0, Paediatrician | 1, Psychologist | 2, Psychiatrist | 3, GP | 4, Occupational therapist | 5, can't recall | 6, Speech pathologist | 7, Other (please specify)]
   b. At what age was [child's name] diagnosed with ADHD/ADD? [0, less than 1 year | 1, 1 year old | 2, 2 years old | 3, 3 years old | 4, 4 years old | 5, 5 years old | 6, can't recall/unsure]
10. Does [child's name] have a diagnosis of global developmental delay, intellectual disability, learning disability or communication or behavioural disorder? (please choose as many options as relevant) [0, No | 1, Yes, global developmental delay | 2, Yes, intellectual disability | 3, Yes, learning disability | 4, Yes, communication disorder | 5, Yes, oppositional defiance disorder (ODD) | 6, Yes, pathological demand avoidance (PDA DMS IV) | 7, Yes, other (please specify)]
   (if yes or seeking diagnosis)
      i. [if yes communication disorder] Specify communication disorder [0, Dyslexia | 1, Language disorder | 2, Speech sound disorder | 3, Social (pragmatic) communication disorder | 4, Childhood onset fluency disorder (stuttering) | 5, unsure]
   b. Who provided this diagnosis? Please check multiple if appropriate. [0, Paediatrician | 1, Psychologist | 2, Psychiatrist | 3, Speech pathologist | 4, Physiotherapist | 5, Occupational therapist | 6, Medical doctor | 7, Other (please specify)]
   c. At what age was [child's name] diagnosed? [0, less than 1 year | 1, 1 year old | 2, 2 years old | 3, 3 years old | 4, 4 years old | 5, 5 years old | 6, can't recall/unsure]
11. Does [child's name] have asthma? [1, Yes | 0, No | 2, seeking diagnosis]
   (if yes or seeking diagnosis)
   a. Who diagnosed/ is involved with diagnosing [child's name] with asthma? [0, Paediatrician | 1, GP | 2, can't recall | 3, Other (please specify)]
   b. At what age was [child's name] diagnosed with asthma? [0, less than 1 year | 1, 1 year old | 2, 2 years old | 3, 3 years old | 4, 4 years old | 5, 5 years old | 6, can't recall/unsure]

**About your family**

1. What is your relationship to the child participating in this study?
   1 Mother
   2 Father
   3 Foster parent
   4 Family relation (e.g. aunt, grandmother)
   5 Other, please specify
2. [if mother] If you were [child's name]'s birth parent, how old were you when [child's name] was born? [text or checkbox 'I was not the birth parent']

3. [if not birth parent according to questions 1 or 2] How old was the mother or birth parent when [child's name] was born? [number]
4. Are you the primary caregiver? [yes, no]
5. [if no] If no, who is the primary caregiver?
    1 Mother
    2 Father
    3 Foster parent
    4 Family relation (e.g. aunt, grandmother)
    5 Other, please specify

6. What is the [child's name]'s family living situation?
    1 Child living with two parents
    2 Child living with one parent
    3 Parents separated/divorced but both have custody rights
    4 Other, please specify
7. How many siblings does [child's name] have?
8. Apart from [child's name], how many children live at home?
9. Please choose the option that best describes [child's name]'s cultural and ethnic background. You may choose as many options as you wish.
    1 Aboriginal
    2 African
    3 East Asian
    4 Hispanic or Latino
    5 Pacific Islander
    6 South Asian
    7 Torres Strait Islander
    8 White/Caucasian
    9 Self-describe

(if self-describe)

Please describe

10. Which of the following best describes your highest level of education?
    1 Left school between 13-16 years
    2 Completed year 11
    3 High school certificate (completed year 12)
    4 University degree
    5 University post graduate degree
11. Do you have other qualifications? (e.g. trade, secretarial) [yes/no]
12. [If yes]: Please specify.
13. Which best describes your employment status at the moment?
    1 Full time paid employment (> 30 hours/week)
    2 Part time paid employment: < 30 hours/week
    3 Volunteer work
    4 Parenting and home duties
    5 Other

14. [If 1,2, or 5], Please provide a title and description of the job

15. Which of the following best describes your household income for the last financial year, before you have paid tax?

1 $0 - $18,200
2 $18,201 - $45,000
3 $45,001 - $120,000
4 $120,001 - $180,000
5 $180,001 -$242,000
6 $242,001 and above

16. Are you currently living with a partner?
    1 Yes
    2 No

[if currently living with a partner]

17. Which of the following best describes your partner's level of education?
    1 Left school between 13-16 years
    2 Completed year 11
    3 High school certificate (completed year 12)
    4 University degree
    5 University post graduate degree

18. Does your partner have other qualifications? (e.g. trade, secretarial) [yes/no]
19. Which best describes your partner's employment status at the moment?
    1 Full time paid employment (> 30 hours/week)
    2 Part time paid employment (< 30 hours/week)
    3 Volunteer work
    4 Parenting and home duties
    5 Other

20. [If 1,2 or 5], Please provide a title and description of the job

21. Who would consider the primary income earner in your household?
    1 Myself
    2 My partner

22. What is the primary language is spoken at home?
    1 English
    2 Combined (English + other language)
    3 Other primary language
23. [if 2 or 3] Please specify other language.

## Additional questions added to the MBQ-C

Options of structured physical activities are informed by the Children's Participation in Organised Physical Activity Outside of School Hours report (https://www.sportaus.gov.au/__data/assets/pdf_file/0004/675562/AusPlay_focus_Children_Participation_2.pdf) and have been further refined using a think aloud interview technique with parents of children born very preterm.

I. This term (or next term if it is currently school holidays), is your child participating in **structured community-based physical activity** on a regular

basis? A structured physical activity is one that is planned and intentionally directed by an adult. Examples include swimming lessons, dance classes or structured multi-sports lessons. Please don't include activities completed during preschool (kinder) or school hours. [yes/no]

II. Please choose from the following list which structured community-based physical activities your child attends. You may choose as many as needed.

- Swimming lessons
- Dance classes
- Gymnastics (including Kindergym)
- Sports class (e.g. Ready Steady Go Kids)
- Athletics (e.g. Little Athletics)
- Soccer
- Circus
- Other

[if other]. Please briefly describe the activity your child attends.

[For 12-months post-baseline only]

III. **(Goal Setting group only)**

A) Did your child start a new community-based physical activity after completing goal setting as part of the Active-Prem study? [yes/no]
B) [If yes]. Please briefly describe the activity (e.g. Kinder Gym).
C) [If yes]. Does your child still attend this activity? [yes/no]

**IV. (Active-Prem group only)**

A) Does your child still attend their 'Active-Prem' community activity? [yes/no]
B) Has your child started any other new community-based physical activities since enrolling in Active-Prem? [yes/no]
C) [if yes to B] Please briefly describe the new activity (e.g. Kinder Gym).
D) [If yes]. Does your child still attend this new activity? [yes/no]

[For post-intervention and 12-months post-baseline only]

V. **(both Active-Prem and Goal Setting groups)**

Over the past 6 months, has your child participated in any research projects that aimed to improve physical activity other than this project [yes/no]
[if yes]. Please describe:

## Barriers and facilitators for supporting children's community-based physical activity participation

These questions have been developed with input from the implementation scientists in our team and further refined through a 'think aloud' interview process with parents involved in our study.

I. What currently makes it difficult to support your child's participation in physical activity? Please include as many things as you can think of.

   a. Please list things that are currently difficult for your child (e.g. becoming short of breath quickly)

   b. Please list things that are currently difficult for you as a parent (e.g. distance from our home)

   c. Please list any other things that make it currently difficult to support your child's participation (e.g. the activity centre hasn't returned my call)

II. [only asked in post-intervention version of survey] Was there anything that was part of the Active-Prem study that made it difficult to support your child's participation in physical activity?

III. What currently makes it easier to support your child's participation in physical activity? Please include as many things as you can think of.

   a. Please list things that make is currently easier for your child (e.g. when they enjoy the activity)

   b. Please list things that make it currently easier for you as a parent (e.g. good timetable)

   c. Please list any other things that make it currently easier to support your child's participation (e.g. my friends are going there)

IV. [only asked in post-intervention version of survey] Was there anything that was part of the Active-Prem study that made it easier to support your child's participation in physical activity?

## Attendance and Smiley Face Scale

1) Did [child's name] attend class today? [yes/no]
2) [if no]. What was the reason [child's name] wasn't able to attend?
   a. My child was unwell
   b. Another family member was unwell

c. Family scheduling or logistics
d. Other reason

3) [if other] Please briefly describe the reason.
4) [if yes to Q1]. Please ask [child's name] what they thought about their class today and record their response below. Please try and ask your child as close as possible to the end of class, as this often gets the most accurate response.

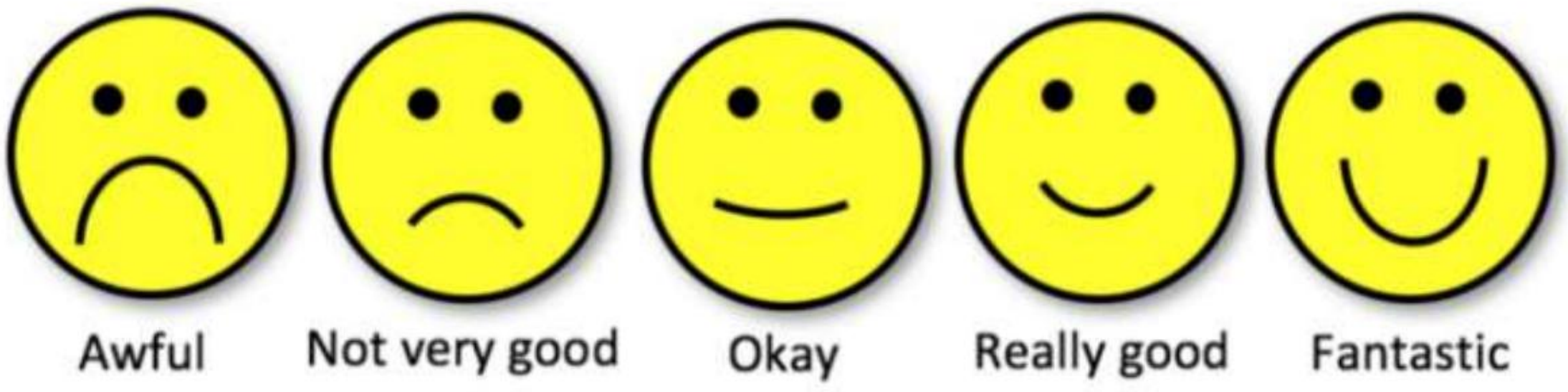


1, Awful

2, Not very good

3, Okay

4, Really good

5, Fantastic

[Introductory session only]

5) Did the introductory session include some time where you and [child's name] could meet the coach, ask questions, and spend some time getting to know each other? [yes/no]
6) [if yes]. Approximately how much time did you, [child's name] and the coach spend getting to know each other? [1, less than 5 minutes | 2, 5-10 minutes | 3, 10-20 minutes | 4, more than 20 minutes]
7) Did the introductory session include some time where [child's name] was able to complete a 'mini class', such as trying out equipment or activities? [yes/no]
8) [is yes] Approximately how time did this 'mini class' take? [1, less than 5 minutes | 2, 5-10 minutes | 3, 10-20 minutes | 4, more than 20 minutes]
9) Is there anything else about the introductory session you would like to share? [optional comment]

## 12-month healthcare utilisation

[delivered 12-months post-baseline]

Families will report their child's health care utilization in the past year using the following questions. A 12-month recall period has been chosen, as this has been shown to produce more accurate recall.

I. Has your child used these health services in the past 12 months? [yes/no]
II. [if yes] Please estimate how many times

| | No. of times |
|---|---|
| Consulted a general practitioner (GP) | |
| Consulted a specialist | |
| Visited hospital emergency department (ED) | |
| Been hospitalized, same day stay | |
| Been hospitalized overnight | |
| -If hospitalized, how many days in total | |
