# Supplementary material for: Enhancing physical activity in early childhood for 3–5-year-old children born very preterm: protocol for an Australian randomised controlled trial of Active-Prem
Source: BMJ Open. 2026 Nov 2;16(11):e124614. doi: 10.1136/bmjopen-2026-124614 (PMC13735262; doi:10.1136/bmjopen-2026-124614)
Supplement: Supplementary file 5 [file bmjopen-16-11-s005.pdf]

Supplementary E: Coach outcome measures developed specifically for the study

Demographic questionnaire

1. Your first name:
2. Your last name:
3. Your pronouns:
4. Post code of location of the physical activity you coach. [post code].
5. Please describe the sport or activity that you coach or teach (e.g. ballet for ages 2-17, junior tennis etc.)[free text]
6. Please detail the days and times of any regularly scheduled classes you coach that are for 3–5-year-olds [free text]
7. How many years have you been a coach or teacher? [number]
8. Did you complete any training or qualifications to coach or teach your activity? [yes/no]
9. [if yes] Please briefly describe the training or qualifications you have completed.[free text]

Fidelity and adaptability survey

Please complete the following survey about the class you coached today that included an Active-Prem participant. If the Active-Prem participant didn't attend class today, please answer based on the most recent class they attended.

Your answers to these questions will help the Active-Prem team understand how we can improve the project in the future to better support coaches.

1) Please describe any **difficulties** you had today in successfully and meaningfully including any child or children enrolled in Active-Prem in your activity.
2) Please describe any strategies or ideas from the *Coaching Essentials: Premature Children in Sport* toolbox that **supported or helped you** to successfully and meaningfully include any child or children enrolled in Active-Prem in your activity.
3) Please describe any **adaptations or work-arounds (beyond those covered in the Coaching Essentials program)** you used today to successfully and meaningfully include any child or children enrolled in Active-Prem in your activity.

4) What should be **included in future versions** of *Coaching Essentials: Premature Children in Sport* that could have supported you today.

Attendance and Involvement scale

1) Did [child's name] attend class today? [yes/no/another coach took class today]
2) [if yes]. How involved was [child's name] in today's class? [5 point Likert scale]
    1) Not very involved
    2)
    3) Somewhat involved
    4)
    5) Very involved

[Introductory session only]

3) Did the introductory session include 'getting to know you' time? [yes/no]
4) [if yes]. Approximately how much time did you and the family spend getting to know each other? [1, less than 5 minutes | 2, 5-10 minutes | 3, 10-20 minutes | 4, more than 20 minutes]
5) Did the introductory session include a tailored introduction to foundational skills? [yes/no]
6) [is yes] Approximately how time did you spend on this activity? [1, less than 5 minutes | 2, 5-10 minutes | 3, 10-20 minutes | 4, more than 20 minutes]
7) Is there anything else about the introductory session you would like to share? [optional comment]
