# Supplementary material for: Enhancing physical activity in early childhood for 3–5-year-old children born very preterm: protocol for an Australian randomised controlled trial of Active-Prem
Source: BMJ Open. 2026 Nov 2;16(11):e124614. doi: 10.1136/bmjopen-2026-124614 (PMC13735262; doi:10.1136/bmjopen-2026-124614)
Supplement: Supplementary file 6 [file bmjopen-2026-124614.reviewer_comments.pdf]

PEER REVIEW HISTORY

BMJ Open publishes all reviews undertaken for accepted manuscripts. Reviewers are asked to complete a checklist review form and are provided with free text boxes to elaborate on their assessment. These free text comments are reproduced below.

ARTICLE DETAILS

Title (Provisional)

Enhancing Physical Activity in Early Childhood for 3-5-year-old Children Born Very Preterm: Protocol for an Australian Randomised Controlled Trial of Active-Prem

Authors

Cameron, Kate; FitzGerald, Tara; Peters , Sanne; Coulston, Free; Binstock, Loni; Mainzer, Rheanna M; Hua, Xinyang; Clark, RA; Cheong, Jeanie LY; Doyle, Lex W; Anderson, Peter J; Lee, Katherine; Dalziel, Kim; Lloyd, Marguerite; Lloyd, Jasmine; Olsen, Joy Elizabeth; Duff, Gemma; Koirala, Prakriti; Kilgour, Gaela; Best, Stephanie; Spittle, Alicia J

VERSION 1 - REVIEW

**Reviewer**	**1**
**Name**	**Mozun, Rebeca**
**Affiliation**	**University Children's Hospital Zürich, Department of Intersive Care and Neonatology and Children's Research Center**
**Date**	**12-Aug-2026**
**COI**	**None**

The authors present a protocol for a type 1 hybrid effectiveness implementation RCT planning to recruit >200 children, aged 3 to 5 years born with <32 weeks of gestational age, which compares the «active pre-term» intervention with «goal setting» as control using 7-day average minutes per day spent stepping as outcome. This manuscript is well written, the topic is of interest, and the methods described are sound. However, some aspects could benefit from further detail and clarification.

A current gap is the lack of statistical analysis plan, which means that methodological and data management aspects from the SPIRIT checklist (provided in the supplement) are not currently detailed (item 6) or are not as detailed as expected and, although the authors mention they plan to make it available before analysis, it is currently not accessible (e.g. item 26). I encourage the authors to include all information requested in the SPIRIT checklist in the manuscript or online supplement, including also items contained in the ethics protocol (item 1b and items in the ethics section).

The recently published SPIRIT-Children and Adolescents 2026 checklist (https://www.bmj.com/content/392/bmj-2025-085062) also recommends to "describe the potential for extrapolation from other paediatric populations or adult data, or why extrapolation is not considered appropriate". Please reflect on this point.

For trials to be representative, minority groups should be included to bring diversity in cultural aspects and perceptions, and to avoid increasing the health gap. How the authors will encourage participation of First Nations communities? Were this group also part of PPIE activities? I would appreciate if the authors could expand on this.

The authors mention they plan to stratify randomization by whether the family has one or more children enrolled in the study (page 19), which I understand they will also accounted for in multilevel analyses. I would be interested in whether they considered stratifying by severity (captured in baseline questionnaires), since this can affect physical activity levels and engagement with the intervention. Please provide a rationale for this.

There are currently no limitations described in the manuscript. Please reflect on these in the strengths and limitations section and in the discussion.

The control group is more likely to have attrition bias at follow-up assessments. How will the authors encourage engagement throughout the trial? Please elaborate on this.

According to the trial registration (https://anzctr.org.au/Trial/Registration/TrialReview.aspx?id=388966), the trial started recruiting already (14.04.2025) and has recruited 68 out of 204 patients. I would suggest mentioning the timeline in the text, and the recruitment status.

It is unclear if the trial recruits only in Melbourne / Victoria (as I initially understood from page 10, recruitment and informed consent) or also in other Australian states and territories (as mentioned in the trial registration). Please clarify this.

The exclusion criteria (page 10) seem quite broad. Who determines if children have a medical condition or procedure that precludes safe participation, and how is this assessed (is there are reference list)?. It is sensible to be cautious when justified, my concern is that children who may need the intervention most are those most affected or with more prematurity sequelae, and may be more likely to be excluded from participation.

How does the DSMB assess trial safety? What data safety is being collected, by whom and how? Are there any interim analysis planned?

The authors plan to assess the difference between groups in the primary outcome using linear regression. Means are more sensible to outliers than medians. Do the authors have preliminary data on the distribution of the outcome to assume it will be normally distributed, or how will they handle analysis if data are skewed?

Minor comments:

- To better understand the rationale for focusing only on children aged 3-5 years and not in older age-groups, please expand if possible.

- The TIDiER description is relevant but lengthy and may disengage readers. Consider moving the full description to the online supplement, and keep in the text more concise key points. Also, the TIDiER acronym needs be indicated when first mentioned in page 9.

- The primary outcome is “minutes spent stepping per day (averaged over 7 days) measured by accelerometry post-intervention” in the text and “7-day accelerometry (average minutes per day spent stepping) at post-intervention” in table 4. I would suggest using the same wording throughout to ensure consistency.

- The abstract could include the location of the trial.

- I thank the authors for providing the questionnaires in the online supplement. I particularly appreciate they will assess socioeconomic status to describe the population. However, I missed a question on whether the participating child has (older) siblings. This may be a factor that motivates them to be more physically active. The authors could consider collecting this information moving forward.

VERSION 1 - AUTHOR RESPONSE

Reviewer comment	Response and corresponding changes (new additions to text are underlined)
Reviewer 1: Dr. Rebeca Mozun, University Children's Hospital Zürich	
The authors present a protocol for a type 1 hybrid effectiveness implementation RCT planning to recruit >200 children, aged 3 to 5 years born with <32 weeks of gestational age, which compares the «active pre-term» intervention with «goal setting» as control using 7-day average minutes per day spent stepping as outcome. This manuscript is well written, the topic is of interest, and the methods described are sound. However, some aspects could benefit from further detail and clarification.	Thank you for your detailed review and comments.
A current gap is the lack of statistical analysis plan, which means that methodological and data management aspects from the SPIRIT checklist (provided in the supplement) are not currently detailed (item 6) or are not as detailed as expected and, although the authors mention they plan to make it available before analysis, it is currently not accessible (e.g. item 26). I encourage the authors to include all information requested in the SPIRIT checklist in the manuscript or online supplement, including also items contained in the ethics protocol (item 1b and items in the ethics section).	We are now using the SPIRIT-C checklist (as you have suggested in your next comment) and have created an additional supplementary document (Supplementary B) to ensure all items in the checklist are included in the protocol paper.<br><br>Any items in the SPIRIT-C checklist that have not been included in the main manuscript, are now included in Supplementary B.<br><br>We have added a sentence to the start of the data analysis section (page 18):<br>A detailed statistical analysis plan (SAP) will be finalised and made publicly available prior to initiating the analysis at the conclusion of the trial. Further details on data management are included in Supplementary B.

	In regard to item 6 and 26 from the SPIRIT checklist, details of data and information management for the study is provided on pages 6-9 of Supplementary B.
The recently published SPIRIT-Children and Adolescents 2026 checklist (https://www.bmj.com/content/392/bmj-2025-085062) also recommends to "describe the potential for extrapolation from other paediatric populations or adult data, or why extrapolation is not considered appropriate". Please reflect on this point.	We have now completed the SPIRIT-C checklist and have included it in our submission.<br><br>We have updated our description: (page 9): This protocol is reported according to the Standard Protocol Items: Recommendations for Interventional Trials<u>- Children and Adolescents</u> (SPIRIT<u>-C</u>) guidelines30<br><br>We have adjusted our introduction to describe the potential for extrapolation (page 5):<br>PA is associated with a range of health and developmental benefits for <u>all</u> children, including better motor skills, greater physical fitness, improved cognitive outcomes, lower rates of chronic disease, and better academic outcomes. [1-3] Furthermore, PA may improve confidence and promote enjoyment and quality of life. [4] Thus, <u>drawing from the broader literature on the benefits of PA for children with and without disabilities,</u> PA offers a multifaceted intervention option for children born VPT given its positive association with outcomes across multiple developmental and health domains that are known to be vulnerable following VPT birth. [5-9]
For trials to be representative, minority groups should be included to bring diversity in cultural aspects and perceptions, and to avoid increasing the health gap. How the authors will encourage participation of First Nations communities? Were this group also part of PPIE activities? I would appreciate if the authors could expand on this.	Whilst our study does not specifically target First Nations groups, they are part of the study population. Our study team, based at The University of Melbourne, is trained in cultural safety and awareness, and how to engage respectfully with First Nations families.<br><br>We have updated our recruitment paragraph (Methods: page 10)<br><br>Potential participants will be asked to complete an online expression of interest form to screen for eligibility. Eligible families will be contacted by a member of the research team, provided with written information about the study and offered an opportunity to discuss the project and ask questions. <u>All members of the research team are trained in cultural safety and awareness to facilitate the inclusion of First Nations families.</u><br><br>Throughout all PPIE activities, we have focused on involving our three key groups of

	knowledge holders; individuals with lived experience of preterm birth, community providers of physical activity and health professionals working with children born very preterm. We have not collected information on the cultural and/or ethnic background of either individuals or members of community groups, organisations and advisory committees.<br><br>Your question has made us realise that we are not currently collecting data on ethic/cultural background of participants in this study. An additional question has now been added to the demographic questionnaire to capture this information (Supplementary D):<br><br>1. Please choose the option that best describes [child's name]'s cultural and ethnic background. You may choose as many options as you wish.<br>1 Aboriginal<br>2 African<br>3 East Asian<br>4 Hispanic or Latino<br>5 Pacific Islander<br>6 South Asian<br>7 Torres Strait Islander<br>8 White/Caucasian<br>9 Self-describe<br>(if self-describe)<br>Please describe
The authors mention they plan to stratify randomization by whether the family has one or more children enrolled in the study (page 19), which I understand they will also accounted for in multilevel analyses. I would be interested in whether they considered stratifying by severity (captured in baseline questionnaires), since this can affect physical activity levels and engagement with the intervention. Please provide a rationale for this.	We didn't stratify by severity as our priority was to stratify by family to ensure that all children in the same family are randomised to the same intervention. This is for practical reasons for families, and because parents of children allocated into the Active-Prem group complete the parent program as part of the intervention. Further stratification by severity could be challenging, as children in the same family could be classified at different severities but randomisation is done at the family level.<br><br>Whilst we appreciate severity can impact on physical activity, we anticipate equal numbers in each treatment arm as it is an RCT.<br><br>We have added a sentence to the randomisation paragraph (page 16-17):<br><br>Randomisation will be stratified by whether the family has one child vs more than one child enrolling in the study, with children in the same

	family randomised to the same group. This approach was chosen so that all children in the same family receive the same intervention and because parents of children randomised to Active-Prem complete the parent program as part of the intervention.
There are currently no limitations described in the manuscript. Please reflect on these in the strengths and limitations section and in the discussion.	A detailed limitation paragraph has now been included in the discussion section (pages 24-5).<br><br>Although Active-Prem has been designed to align with the needs and preferences of children born VPT and their families, with potential to be implementable on a larger scale if found effective, there are some limitations to this study. Children with sensory or behavioural difficulties may find it challenging to wear the accelerometer continuously for 7 days, which may impact data collection completion for our primary outcome measure. Another limitation is the complexity of the Active-Prem intervention; completion of all six steps may be delayed or impacted by potential difficulty recruiting coaches in a timely manner while engagement with parent support (step 2) and coach training (step 4) may differ among participants. Furthermore, both the coach and parent programs are only available in English, which limits the linguistic diversity of the sample. Finally, although we have chosen to compare two active interventions, rather than intervention and control, the Goal Setting group have less interaction with the study, and therefore may be more likely to disengage. We are trying to mitigate this limitation by providing families with feedback on their child's PA at two timepoints throughout the study to support engagement from both groups.
The control group is more likely to have attrition bias at follow-up assessments. How will the authors encourage engagement throughout the trial? Please elaborate on this.	To minimise attrition, we chose to have an active comparison (goal setting group) rather than a control group. For this group, we try to keep them engaged through two reports, which include details on their child's physical activity compared with the Australian 24-hour-movement-guidelines.<br>We have added to the discussion section (page 25)<br><br>Finally, although we have chosen to compare two interventions, rather than intervention and control, the Goal Setting group have less interaction with the study, and maybe therefore be more likely to disengage. Providing families with feedback on their child's PA at two

	timepoints throughout the study is designed to support engagement from both groups.
According to the trial registration (https://anzctr.org.au/Trial/Registration/TrialReview.aspx?id=388966), the trial started recruiting already (14.04.2025) and has recruited 68 out of 204 patients. I would suggest mentioning the timeline in the text, and the recruitment status.	We have added the following sentence to the recruitment and informed consent paragraph (page 10).<br><br>Recruitment for Active-Prem commenced in April 2025, and is planned to be completed by September 2027.
It is unclear if the trial recruits only in Melbourne / Victoria (as I initially understood from page 10, recruitment and informed consent) or also in other Australian states and territories (as mentioned in the trial registration). Please clarify this.	The recruitment paragraph has been updated to make it clear that participants will be recruited across Australia (page 10):<br><br>**Recruitment and informed consent**<br>Recruitment for Active-Prem commenced in April 2025, and is planned to be completed by September 2027. Recruitment was initially restricted to the state of Victoria, Australia. However, in November 2025, recruitment was expanded to Australia-wide.<br><br>**Parent-child dyads**: Families will be recruited from across Australia via advertising through our partner organisations (e.g. Life's Little Treasures Foundation and Miracle Babies who support families of children born preterm), through organisations affiliated with our research team members (The University of Melbourne, The Royal Children's Hospital, Royal Women's Hospital and Murdoch Children's Research Institute), sharing advertising materials amongst clinical Australian paediatric physiotherapists, and social media platforms that target parents of children in early childhood.
The exclusion criteria (page 10) seem quite broad. Who determines if children have a medical condition or procedure that precludes safe participation, and how is this assessed (is there are reference list)?. It is sensible to be cautious when justified, my concern is that children who may need the intervention most are those most affected or with more prematurity sequelae, and may be more likely to be excluded from participation.	We agree that we do not want to unnecessarily exclude children who may benefit from support with physical activity. The approach we take is designed to include as many children as possible, while ensuring safety.<br><br>We have added a paragraph to the exclusion section to expand upon this approach (page 9)<br><br>This study aims to recruit as inclusively as practicable. The focus is on safety rather than excluding children with medical conditions who may require guidance from a healthcare professional to participate. For children whose parent/carer indicate that their child may not be safe to participate, a member of the study team will (with the permission of the parent/carer) seek the opinion of the child's treating medical

	professional as to the safety of the child's participation. Written permission will be sought from treating healthcare professionals in each of these cases. For children who have a medical procedure planned, the study team will seek permission to re-contact the family at a later date (if the study timeline allows) to re-evaluate eligibility.
How does the DSMB assess trial safety? What data safety is being collected, by whom and how? Are there any interim analysis planned?	The DSMB will meet annually to assess study progress, protocol compliance, data quality and trial safety, and provide recommendations to the study team (e.g. study continue as is, be modified or is stopped).<br><br>We have moved information on the DSMB to supplementary material for word count reasons. The following is included on page 23:<br>Safety oversight will be under the direction of an independent Data and Safety Monitoring Board (DSMB). Further details on the DSMB, trial management, risk and safety are provided in Supplementary B.<br><br>The following has been included in Supplementary B.<br><br>The safety data that are being collected as part of this trial and that will be included in the DSMB report are; adverse events, related adverse events, serious adverse events, unexpected, related serious adverse events, significant safety issue and urgent safety measures. The reporting of safety events for this study follows the Australian National Health and Medical Research Council's 'Safety monitoring and reporting in clinical trials involving therapeutic goods' (November 2016). DSMB will provide its input to the Sponsor-Investigator. Further details on trial management, risk and safety is provided in Supplementary B.<br><br>Trial safety data is being collected throughout the trial period for each participant. Parents/carers and coaches are asked to contact the study team via phone call or email if any adverse events occur during the intervention. For the purposes of this trial, Adverse Events will be recorded if they are possibly, probably or definitely related to the intervention (or may be partially related to the intervention) or will impact the ongoing participation of the participant in the intervention (i.e. the participant may discontinue classes if they become unwell). Adverse events in this context

	may include musculoskeletal injury. Conditions that are present during screening, and do not deteriorate, will not be considered adverse events. The adverse event will be described in an Expedited Safety Form.<br><br>There are no interim analyses of the efficacy outcomes planned for this trial. We have indicated in on page 18 that the analysis will take place at the conclusion of the trial:<br><br><u>A detailed statistical analysis plan (SAP) will be finalised and made publicly available prior to initiating the analysis at the conclusion of the trial. Further details on data management are included in Supplementary B.</u>
The authors plan to assess the difference between groups in the primary outcome using linear regression. Means are more sensible to outliers than medians. Do the authors have preliminary data on the distribution of the outcome to assume it will be normally distributed, or how will they handle analysis if data are skewed?	From physical activity data from previous studies conducted by our research team (FitzGerald et. al., 2021; Cameron et. al., 2025) and from data collected to date for this study, stepping physical activity data is normally distributed. We have therefore chosen to keep our analysis plan as is (page 18-19).<br><br>Analysis of primary objective: The primary estimand of interest is the mean difference in average minutes spent stepping per day measured by accelerometry during the post-intervention period, between the Active-Prem and Goal Setting groups, using a treatment policy approach (all participants will be analysed according to their treatment allocation irrespective of whether or not they received any of the intervention) to handle the intercurrent event of non-compliance to the intervention.34 . This will be estimated using linear regression, adjusted for multiple birth. The model will be fitted using generalised estimating equations to account for the correlation between siblings (e.g., twins) within families and robust (sandwich) standard errors will be used to estimate the 95% confidence interval (CI).
Minor comments:<br>To better understand the rationale for focusing only on children aged 3-5 years and not in older age-groups, please expand if possible.	The following is included in our introduction to explain the rationale (pages 5-7):<br><br>At a group level, children born VPT in early childhood (aged 3-5 years) are less physically active than their term-born peers, [10] with 50% not meeting Australian 24-hour physical activity (PA) guidelines. [10, 12, 13]<br><br>Active-Prem is a novel intervention built on substantial preliminary research into

	understanding and improving PA participation for 3-5-year-old children born VPT, and addresses an expressed need from parents of children born VPT for support with PA participation for this age group. [10, 12-17]
The TIDiER description is relevant but lengthy and may disengage readers. Consider moving the full description to the online supplement, and keep in the text more concise key points. Also, the TIDiER acronym needs be indicated when first mentioned in page 9.	While we appreciate that the TIDiER description is long, we feel it important to include in the main text, as it is the description of the intervention being tested, providing rigour and potential for replication. To improve readability, we have re-formatted so that part of the TIDieR is in table format. We have also edited for word count so that many sections are now shorter.<br><br>Updated: New table 2.<br>Numbers of tables 2-5 adjusted.<br><br>Beginning of intervention description now reads:<br><br>The Active-Prem intervention comprises six key steps (Figure 1), each described using the TIDiER format: items 1-3 (name, rationale and 'what') are described below, while the remaining items ('who', 'how', 'where', 'when and how much' and 'tailoring') are detailed in Table 2.[31]<br><br>A definition for the acronym has been added on page 9:<br>the Template for Intervention Description and Replication (TIDiER) checklist
The primary outcome is "minutes spent stepping per day (averaged over 7 days) measured by accelerometry post-intervention" in the text and "7-day accelerometry (average minutes per day spent stepping) at post-intervention" in table 4. I would suggest using the same wording throughout to ensure consistency.	The description of the primary outcome in Table 5 (previously table 4) has now been updated to match the text and now reads:<br><br>Objectively measured PA measured: minutes spent stepping per day (averaged over 7 days) measured by accelerometry post-intervention
The abstract could include the location of the trial.	We have added this information to the introduction section of the abstract:<br>This paper describes the protocol for a randomised controlled trial (RCT) evaluating the effectiveness of Active-Prem (a 6-step intervention combing parent support, coach training and a term of physical activity) compared with Goal Setting, at improving PA for children born VPT living in Australia.
I thank the authors for providing the questionnaires in the online supplement. I particularly appreciate they will assess socioeconomic status to describe the population. However, I missed a question on whether the participating child has (older) siblings. This may be a	In response to your comment, we have added two questions about siblings.<br><br>This has been updated in the Supplementary D:

factor that motivates them to be more physically active. The authors could consider collecting this information moving forward.	2. How many siblings does [child's name] have?<br>3. Apart from [child's name], how many children live at home?

FitzGerald TL, Cameron KL, Albesher RA, Mentiplay BF, Lee KJ, Clark RA, et al. Strength, Motor Skills, and Physical Activity in Preschool-Aged Children Born Either at Less Than 30 Weeks' Gestation or at Term. Physical Therapy. 2021;101(5).
Cameron KL, Mainzer RM, Olsen JE, Clark RA, Cheong JLY, FitzGerald TL, Spittle AJ. Community participation, physical activity, and quality of life for children born very preterm. Dev Med Child Neurol. 2025;67(10):1331-9.

VERSION 2 - REVIEW

Reviewer 1

Name Mozun, Rebeca

Affiliation University Children's Hospital Zürich, Department of Intersive Care and Neonatology and Children's Research Center

Date 14-Oct-2026

COI

I thank the authors for considering and implementing my comments, and am happy with the current version.
